# The Gut–Brain Axis in Fetal Alcohol Spectrum Disorder (FASD): Why the Gut Shapes Behavior, Depression, and Self-Injurious Behavior in Children with Prenatal Alcohol Exposure—A Narrative Review with a Proposal for Staged Nutritional and Microbiological Intervention

**DOI:** 10.3390/jcm15166390

**Published:** 2026-08-18

**Authors:** Katarzyna Zych-Krekora, Oskar Sylwestrzak, Michał Krekora

**Affiliations:** 1 Department of Perinatology, Obstetrics and Gynecology, Polish Mother’s Memorial Hospital Research Institute (Instytut Centrum Zdrowia Matki Polki, ICZMP), 93-338 Łódź, Poland; sylwestrzakoskarpatryk@gmail.com (O.S.); krekoram@poczta.onet.pl (M.K.); 2 Faculty of Medicine, University of Social Sciences (Społeczna Akademia Nauk, SAN), 90-113 Łódź, Poland

**Keywords:** FAS, FASD, prenatal alcohol exposure, gut microbiota, gut–brain axis, short-chain fatty acids, sodium butyrate, psychobiotics, depression, self-injurious behavior, ADHD, underdiagnosis, ethyl glucuronide, foster care, co-exposure, preterm birth, small for gestational age, nutritional interventions, choline, DHA, ARA

## Abstract

Prenatal alcohol exposure (PAE) leads to fetal alcohol spectrum disorder (FASD), the most common preventable cause of neurodevelopmental impairment. The classical narrative attributes the clinical picture of FASD exclusively to direct ethanol-induced brain injury. In the present review, we argue that this perspective is incomplete and leads to diagnostic errors, most often to the misdiagnosis of ADHD in children who in fact have FASD. We propose that, alongside the direct neurotoxicity of ethanol, an important and clinically under-recognized complementary mechanism is gut–brain axis dysfunction: alcohol damages the enteric nervous system and enteric glial cells, induces dysbiosis with deep deficits of butyrate and other short-chain fatty acids (SCFAs), damages the enterochromaffin cells responsible for 90% of peripheral serotonin production, and—through translocation of lipopolysaccharide (LPS) and activation of the Toll-like receptor 4 (TLR4)—sustains a neuroinflammatory brain signature. This cascade—superimposed on direct ethanol neurotoxicity—may account for the high rates of depression, anxiety, self-injurious behavior, and suicide attempts observed in individuals with FASD and for the limited efficacy of traditional interventions focused solely on the central nervous system. The 2024 Polish Institute of Mother and Child (Okulicz-Kozaryn et al.) study showed that 50.3% of pregnant women consumed alcohol, and 11% did so regularly, against only 7% who admitted so in questionnaires. The real clinical picture of children with FASD is further complicated by three factors to which we devote separate sections in this paper: prenatal co-exposure to nicotine, cannabinoids, and opioids; the loss of vertical microbiota transmission and breastfeeding in children transferred to foster care (where the prevalence of FASD is 18.8% and in children’s homes in some regions reaches up to 80%); and the substantial over-representation of preterm and small-for-gestational-age (SGA) infants (in the Hasken et al. cohort, 18.4% of children with FASD were born preterm and 51.4% were born SGA). In the final section, we present a structured, staged protocol for nutritional and microbiological intervention grounded in a hierarchy of evidence: from interventions supported by randomized controlled trials (RCT-level; choline) through interventions supported by strong mechanistic rationale and RCTs in related populations (sodium butyrate, *Lactobacillus rhamnosus* GG, GOS/FOS prebiotics—galacto-oligosaccharides and fructo-oligosaccharides, and omega-3 fatty acids) to experimental interventions. The protocol also covers the window before 2 years of age: we argue that, given the over-representation of preterm and SGA infants among children with FASD, the analogy to preterm infants on parenteral nutrition and to post-institutional infants applies in substantial part to the same patients, which justifies extending the indications for choline and other nutritional interventions. The paper includes a compact table of dosing proposals for each age window (from pregnancy to school-age child) and provides clinicians with concrete answers: where to start, what to avoid, and what to monitor, with explicit signposting of regulatory limitations for individual substances in Poland and the European Union.

## 1. Introduction: Why the Brain Alone Is Not Enough

Prenatal alcohol exposure (PAE) is the most common preventable cause of neurodevelopmental disability. A global meta-analysis by Lange et al. indicates that fetal alcohol spectrum disorder (FASD) affects approximately 7.7 per 1000 children in the general population and up to 113 per 1000 in some European populations [1]. The worldwide prevalence of alcohol consumption during pregnancy is approximately 9.8%, reaching 25.2% in the European region [2]. Despite this, FASD remains one of the most poorly recognized neurodevelopmental disorders in pediatrics. In the study by Chasnoff, Wells, and King, which included 547 children who had been adopted or placed in foster care, FASD was correctly diagnosed in only 13.5% of cases, whereas 86.5% of the children were left undiagnosed or received an incorrect diagnosis: in the vast majority of cases, a diagnosis of ADHD [3].

This diagnostic gap is not neutral. In their classical analysis of 415 individuals with FASD, Streissguth et al. showed that 94% of patients meet the criteria for a psychiatric disorder at some point in life, 23% of adults attempt suicide (five times the U.S. population mean), and rates of depression, addiction, contact with the criminal justice system, and loss of the capacity for independent living are several-fold higher than in the general population [4]. In an Australian population, Tan et al. confirmed that the risk of suicidal behavior in individuals with FASD is substantially increased, and the presence of concurrent depression raises this risk fourfold (OR = 4.20) [5].

The classical explanation of these observations invokes the direct neurotoxicity of ethanol: reduced brain volume, disturbances in myelination, and cortical disorganization. This explanation is valid but incomplete. Over the past decade, a growing body of evidence has indicated that an important, clinically under-recognized complementary mechanism is **gut–brain axis dysfunction**. Ethanol does not act only on the developing brain: it also impairs the function of the enteric nervous system, the shaping of the microbiota, the integrity of the gut barrier, and tryptophan metabolism. These changes may contribute substantially to the behavioral, emotional, and psychiatric phenotype observed in FASD, in parallel with the classical structural changes in the central nervous system.

The present review has four aims: 1. to explain why the gut–brain axis is important in FASD and how specific mechanisms in the gut translate into depression, self-injurious behavior, and ADHD-like symptoms; 2. to describe the scale of underestimation of prenatal alcohol exposure in Poland in light of new data from the Institute of Mother and Child (IMiDz, Instytut Matki i Dziecka, Warsaw) and to show how it translates into diagnostic errors; 3. to account for three factors specific to the real-world population of children with FASD: **prenatal co-exposure to other substances**; **loss of breastfeeding and vertical microbiota transmission in children placed in foster care**; and **preterm birth and small-for-gestational-age (SGA) status as common concurrent consequences of PAE**; and 4. to propose a **structured, staged nutritional and microbiological intervention protocol**—for whom to start, with which active substance, at which dose, and what to monitor—covering the prenatal period, infancy (<2 years), and the preschool/school-age period.

The central practical tool of this review is Table 1, which summarizes the proposed interventions, doses for each age window, the strength of the clinical rationale, and the key publications.

## 2. Methodology of the Literature Review

The present work is a narrative review. Given the high standards of transparency required by high-impact biomedical journals, we adopted reporting principles consistent with the SANRA recommendations (*Scale for the Assessment of Narrative Review Articles*) [34], although a formal PRISMA protocol is not required for this type of publication.

### 2.1. Databases and Search Strategy

The literature search was performed in five databases: **PubMed/MEDLINE**, as the primary source of biomedical literature with access to MeSH-indexed peer-reviewed publications; **Scopus**, for interdisciplinary coverage and citation tracking; **Embase**, for European literature and publications not indexed in MEDLINE; **Web of Science**, for quality assessment and citation tracking in highly indexed journals; and **Google Scholar**, for the gray literature and guidelines from scientific organizations.

During the review process, in response to reviewer comments, the search was extended retrospectively to include Embase and Web of Science. This extension confirmed that the databases originally used (PubMed and Scopus) provided full coverage of the key publications while also enabling the identification of several supplementary publications that were incorporated into the present version of the manuscript.

The **CINAHL** (nursing sciences) and **PEDro** (physiotherapy) databases were not included because the thematic scope of the present review covers medical interventions (pediatric, neonatal, and gastroenterological) and psychiatric interventions rather than nursing or physiotherapeutic ones. The exclusion of these databases is consistent with the defined scope of the work and is acknowledged as a deliberate methodological limitation.

### 2.2. Search Terms

The search strategy was based on combinations of MeSH terms and free-text words, covering: (i) the population: “fetal alcohol spectrum disorder”, “prenatal alcohol exposure”, “FASD”, and “PAE”; (ii) the mechanism: “gut–brain axis”, “microbiota”, “intestinal barrier”, “short-chain fatty acids”, “SCFA”, “butyrate”, “tryptophan metabolism”, “kynurenine pathway”, “neuroinflammation”, and “TLR4”; (iii) interventions: “choline supplementation”, “phosphatidylcholine”, “glycerophosphocholine”, “DHA”, “arachidonic acid”, “prebiotics”, “GOS”, “FOS”, “probiotics”, “*Lactobacillus rhamnosus*”, “lactoferrin”, “bovine colostrum”, and “sodium butyrate”; (iv) subpopulations: “infants”, “preterm infants”, “small for gestational age”, and “foster care”. Terms were combined with Boolean operators.

### 2.3. Inclusion and Exclusion Criteria

**Included** were publications meeting the following criteria: randomized controlled trials (RCTs), systematic reviews and meta-analyses, prospective and retrospective cohort studies, mechanistic animal studies, high-quality narrative reviews from the last 5 years, and official clinical, regulatory, and safety documents (ESPGHAN, EFSA, WHO, the Polish Society of Gynecologists and Obstetricians, EU Regulation 2016/127, and United States Food and Drug Administration safety communications). Date range: January 2010 to December 2025, extended during revision to include documents published or updated in 2026 that supersede earlier guidance [15,35]. Publications predating 2010 were included where they remain the primary source for a specific claim: Streissguth et al. [4], Moro et al. [27], Arslanoglu et al. [28], Scholtens et al. [36], Bruzzese et al. [37], Barker [38], and Manzoni et al. [17]. Languages: English and Polish. Study population: humans (all age groups, with particular emphasis on preterm infants, infants, and children) or animal models directly relevant to the mechanism.

**Excluded** were conference abstracts without a full text; publications in non-peer-reviewed sources (with the exception of guideline and regulatory documents); case reports lacking mechanistic or epidemiological value; animal studies not directly related to the gut–brain axis or FASD; and publications on gut–brain disorders in adults without implications for the pediatric population.

### 2.4. Rationale for the Date Range and Language Choice

The 2010–2025 window reflects the period during which the modern concept of the microbiota–gut–brain axis as a relevant neurodevelopmental mechanism was established: Erny et al. [39]: the microbiota as a regulator of microglial maturation and function; Yano et al. [40]: regulation of serotonin biosynthesis by the microbiota; Pascual et al. [41]: the TLR4 mechanism specific to FASD; and Cryan et al. [42]: a comprehensive review of the microbiota–gut–brain axis. Earlier publications of foundational importance were included as exceptions to this rule.

The language restriction to English and Polish is justified by three considerations: (i) English-language publications account for more than 95% of the current literature on FASD and the gut–brain axis; (ii) the inclusion of Polish is essential in order to cover local epidemiological data (the Okulicz-Kozaryn et al. cohort from IMiDz), clinical data (studies by Dyląg et al. concerning the Polish population with FASD), and regulatory data (the foster care system and Polish guidelines); and (iii) mechanistically important publications in other languages are typically indexed together with English translations or cited in meta-analyses that were included in the review. The exclusion of other languages is acknowledged as a deliberate limitation in Section 14.1.

### 2.5. Assessment of the Quality of the Literature

To assess the strength of evidence of each cited publication, we used the Oxford Centre for Evidence-Based Medicine (CEBM) Levels of Evidence 2011, adapted to the needs of a review article. The evidence levels are indicated in Table 1 and in the discussion text for individual mechanistic and clinical statements. A distinction between evidence from FASD-specific RCTs (Tier 1), evidence extrapolated from related populations (Tier 2), and mechanistic rationale without RCT validation (Tier 3) is explicitly labeled next to each therapeutic recommendation.

### 2.6. Data Selection and Synthesis

The selection of the literature and the extraction of key information were performed independently by two authors (KZK and OS). Disagreements in the classification of publications were resolved by consensus, with the involvement of a third author (MK). The choice of supplementary publications during the review process was made according to the same principles.

## 3. The Scale of the Problem in Poland: The Institute of Mother and Child Study and the Myth of “Women Who Do Not Drink”

For decades, Polish epidemiology of PAE was based mainly on questionnaire studies. Figures from the Polish State Agency for the Prevention of Alcohol-Related Problems, of the order of 30% of pregnant women declaring some alcohol consumption, circulated widely in this period; we cite them here only as the questionnaire-based background against which the biomarker data are set and not as a verified estimate, since no primary agency document could be identified for them. A cross-sectional study conducted at the Department of Obstetrics and Gynecology of the Institute of Mother and Child (IMiDz) in Warsaw, led by Katarzyna Okulicz-Kozaryn, changed this narrative radically [43].

The study included 150 women, from whom hair samples were obtained after delivery and analyzed for ethyl glucuronide (EtG), a direct, indisputable biomarker of alcohol consumption that accumulates in hair in proportion to exposure over the preceding months. The results were striking:

- **A total of 50.3% of the women** had detectable EtG, indicating alcohol consumption during pregnancy;
- **Approximately 11% of the women** had EtG concentrations exceeding 30 pg/mg, indicating regular consumption of substantial amounts of alcohol;
- **Only 7% of the women** admitted alcohol consumption in questionnaires administered in the same group.

The gap between what women report and what the biomarker detects is sevenfold. In other words, for every pregnant woman who admits to drinking, seven drank but did not report it. In addition, the same study showed that alcohol consumption during pregnancy was associated with a **sixfold higher risk of delivering a small-for-gestational-age (SGA) infant**, a finding that reinforces the argument developed in Section 9.3, namely that the population of children with FASD overlaps substantially with the populations of preterm and SGA infants.

The consequences of this discrepancy are twofold, and both are serious: 1. **Underestimation of prenatal exposure**: If the actual PAE in Poland is ~50%, the expected prevalence of FASD is substantially higher than the 7.7/1000 reported in the Lange meta-analysis, possibly closer to the European data from high-risk populations (40–110/1000). 2. **Underestimation of exposure in an individual child**: When a pediatrician sees a child with behavioral difficulties, hyperactivity, and attention deficits, the maternal history is likely to include “I did not drink during pregnancy”, with more than 80% probability that this statement is untrue.

This brings us to the other side of the same problem: the overdiagnosis of ADHD.

## 4. ADHD or FASD? The Mechanism of Misdiagnosis

The clinical features of FASD and ADHD overlap to a degree that is indistinguishable to an untrained clinician. Difficulty sustaining attention, impulsivity, hyperactivity, executive function problems, disorganization, and working memory deficits: all of these features are present in both disorders. In the Chasnoff study cited above, most children ultimately diagnosed with FASD had initially been referred with a diagnosis of ADHD [3]. In the German cohort of Ehrig et al., 256 of 694 children (37%) referred with ADHD symptoms ultimately met the criteria for FASD [44].

Why does this matter clinically, if the symptoms are similar?

**First, the treatment is not the same.** Stimulants (methylphenidate and amphetamine)—the first-line agents in ADHD—are considerably less effective in FASD than in “classical” ADHD and more often cause adverse effects. This is because the attention deficit in FASD has a different neurochemical basis (serotonergic and kynurenine disturbances, neuroinflammation, and microglial dysfunction) than in ADHD, which is based primarily on disturbances of the dopaminergic–noradrenergic system.

**Second, the psychiatric risk is substantially higher in FASD.** Individuals with ADHD without FASD do not carry a 23% lifetime risk of a suicide attempt in adulthood. Individuals with FASD do. A misdiagnosis deprives the child of access to specialized multidisciplinary interventions, FASD-focused care, and family support.

**Third, generational prevention is important.** The diagnosis of FASD in a child is a signal to assess drinking patterns in the family, to provide education to younger siblings, and—in the case of an adolescent girl—to prevent PAE in her own future pregnancy. A diagnosis of ADHD does not trigger any of these mechanisms.

At the intersection of these two problems—underestimation of PAE and phenotypic overlap between FASD and ADHD—lies a hypothesis that can now be confirmed mechanistically: **the “ADHD-like” symptoms in some of these children arise not from dysfunction of the dopaminergic prefrontal cortex but from dysregulation of the gut–brain axis**. We develop this hypothesis in the following sections.

### A Practical Diagnostic Aid: An Algorithm to Support Clinical Decision Making in Primary Care

In Polish clinical practice, where the maternal history for alcohol consumption during pregnancy is misreported in more than 80% of cases [43], the primary care physician assessing a child with suspected ADHD needs a tool to support the clinical decision to refer the child for multidisciplinary FASD diagnostics.

We propose an adaptation of the **FASDetect** algorithm developed and validated by Ehrig et al. in a cohort of 445 children from a German university referral clinic (275 with FASD and 170 with isolated ADHD) [44]. The algorithm is based on a random forest model using **six clinical variables that are readily available in outpatient practice**: (1) birth length; (2) head circumference at birth; (3) IQ (measured by a standardized test: Wechsler, Stanford–Binet, or equivalent); (4) socially intrusive behavior (assessed by the CBCL scale); (5) poor memory (clinical assessment based on caregiver interview and school information); and (6) sleep disturbances (sleep diary and caregiver interview).

The random forest model based on these six variables achieves a **cross-validated AUC = 0.92 (95% CI, 0.84–0.99)** in distinguishing FASD from ADHD in youth with ADHD symptoms, which represents very high diagnostic accuracy comparable with the full 13-variable model [44]. The FASDetect application is publicly available and can be used directly by primary care physicians.

**Practical recommendation.** We propose the use of the FASDetect algorithm as **first-line decision support** in any child who meets both of the following criteria: (i) symptoms suggestive of ADHD of clinically important severity and (ii) at least one of the following: adoption or foster-care placement; uncertain or denied maternal history of alcohol consumption during pregnancy; presence of facial dysmorphic features typical of FAS; microcephaly; or a history of intrauterine growth restriction or preterm birth without another established etiology. A positive FASDetect result should prompt referral to a multidisciplinary diagnostic team (pediatrician + pediatric neurologist + psychologist + child psychiatrist). As an additional practical resource, the AAP Flow Diagram for Medical Home Evaluation of FASD can be used [45].

**Figure 1:**
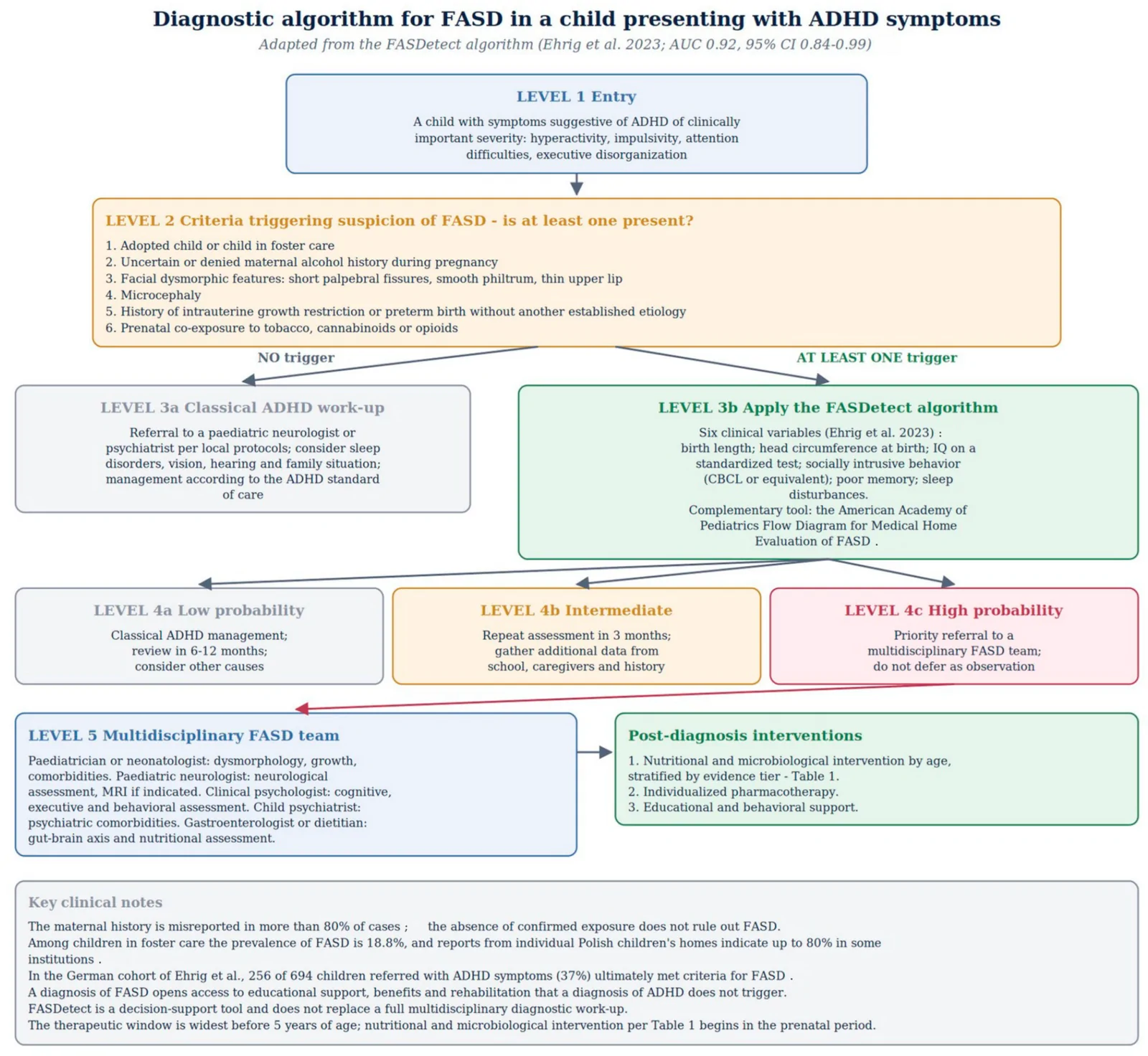
Diagnostic algorithm for FASD/ADHD in primary care, adapted from the FASDetect algorithm (Ehrig et al. 2023 [44], AUC = 0.92; 95% CI, 0.84–0.99). The algorithm proceeds in five levels: (1) entry–a child with symptoms suggestive of ADHD of clinically important severity; (2) six criteria triggering suspicion of FASD (adoption/foster care; uncertain or denied maternal alcohol history during pregnancy; facial dysmorphic features; microcephaly; history of intrauterine growth restriction or preterm birth without another established etiology; prenatal co-exposure to tobacco, cannabinoids, or opioids); (3) branching–with no triggers, classical ADHD management; with at least one trigger, application of the FASDetect algorithm (six clinical variables: birth length, head circumference, IQ, socially intrusive behavior, poor memory, and sleep disturbances); (4) result-driven pathway–low, intermediate, or high probability of FASD; and (5) multidisciplinary team assessment (pediatrician/neonatologist, pediatric neurologist, clinical psychologist, child psychiatrist, and optional gastroenterologist/dietitian) with linkage to the intervention protocol in Table 1. Abbreviations: FASD–fetal alcohol spectrum disorder; ADHD–attention-deficit/hyperactivity disorder; PAE–prenatal alcohol exposure; SGA–small for gestational age; AUC–area under the ROC curve; CI–confidence interval; CBCL–Child Behavior Checklist; MRI–magnetic resonance imaging; AAP–American Academy of Pediatrics; PCP–primary care physician. Sources cited within the figure: misreported maternal history [43]; Ehrig et al. 2023 (FASDetect) [44]; AAP Flow Diagram [45]; prevalence in foster care [46].

The algorithm proposed for Polish clinical practice is presented graphically in Figure 1.

## 5. Ethanol Hits the Gut in Four Places at Once

Ethanol and its toxic metabolite, acetaldehyde, cross the placenta freely and reach fetal concentrations close to maternal levels. Tissues with high water content—including the developing brain but also the enteric nervous system—take it up particularly readily. The direct consequences in the gut include four parallel mechanisms.

### 5.1. Damage to the Enteric Nervous System (ENS) and Enteric Glial Cells (EGCs)

The enteric nervous system, comprising approximately 200–600 million neurons, develops from the same neural crest cell populations as the central nervous system. Ethanol induces apoptosis of these precursor cells, leading to a persistent reduction in the number of enteric neurons and glial cells in the offspring [47]. Enteric glial cells (EGCs) serve functions analogous to those of astrocytes in the brain: they regulate neuronal activity, modulate the immune response in the mucosa, and maintain the integrity of the gut barrier.

### 5.2. Damage to Enterochromaffin Cells: Lost Serotonin

The enterochromaffin cells (ECs) of the gut are the source of **approximately 90% of peripheral serotonin** in the body [40]. These specialized neuroendocrine cells synthesize 5-hydroxytryptamine (5-HT) from tryptophan, and their activity is directly regulated by microbial metabolites, in particular spore-forming bacteria of the *Clostridiaceae* family and short-chain fatty acids produced by *Lachnospiraceae* and *Ruminococcaceae*. In a landmark paper in *Cell*, Yano et al. showed that germ-free mice have gut and blood serotonin concentrations reduced by ~60% and that administration of SCFAs and metabolites from spore-forming bacteria restores 5-HT production.

In FASD, two phenomena weaken this system simultaneously: alcohol acts directly and toxically on ECs during their differentiation, and the alcohol-induced dysbiosis reduces the number of SCFA-producing bacteria. The net effect is a **persistently reduced production of peripheral serotonin**.

### 5.3. Dysbiosis and the Loss of Butyrate Producers

In a rat model, Bodnar et al. showed that alcohol consumption during pregnancy produces a characteristic and persistent dysbiotic profile in the offspring [47]: a decrease in butyrate-producing bacteria (*Lachnospiraceae NK4A136 group* and *Ruminococcus*); an increase in pro-inflammatory taxa (*Parabacteroides*, *Alistipes*, and *Enterobacterales*, including *Salmonella*); reduced overall diversity; and disrupted vertical microbiota transmission from mother to offspring.

In the developmental study by Vella et al., butyrate concentrations in PAE offspring were reduced at the time of weaning (P22), and this reduction was accompanied by an increase in pro-inflammatory cytokines in the amygdala and hypothalamus, structures directly responsible for the regulation of emotions [48].

### 5.4. A Leaky Gut Barrier and LPS Translocation

Butyrate is the main energy source for colonocytes and at the same time regulates the expression of tight-junction proteins (ZO-1, occludin, and claudins). Butyrate deficiency weakens these junctions, resulting in **increased permeability of the gut barrier** (“leaky gut”). Lipopolysaccharide (LPS) translocates through the leaky barrier into the systemic circulation, activating Toll-like receptor 4 (TLR4, *Toll-like receptor 4*) on microglia and inducing the release of TNF-α, IL-6, and IL-1β.

A key mechanistic finding: In *Tlr4*-knockout mice, prenatal alcohol exposure **did not produce** the characteristic neurodevelopmental or behavioral abnormalities [41]. Without TLR4, there is no neuroinflammatory FASD phenotype.

### 5.5. FASD Within the Fetal Programming Framework

The mechanisms described in Section 5.1, Section 5.2, Section 5.3 and Section 5.4 fit within the broader conceptual framework of **fetal programming** (Developmental Origins of Health and Disease, DOHaD). According to this framework, originally formulated by Barker and developed over the past two decades, environmental exposures in the prenatal and early infant periods permanently program the structure and function of physiological systems, shaping the health trajectory and disease susceptibility across the life course [38].

In FASD, prenatal ethanol exposure activates the developmental programming mechanism along multiple pathways: (i) through **direct epigenetic effects**: DNA methylation, histone modifications, and dysregulation of non-coding RNAs [49]; (ii) through **programming of the neonatal gut microbiota**: mode of delivery, feeding (breast milk vs. formula), perinatal antibiotic exposure, and maternal nutritional status determine the composition of the initial microbiota, which, in turn, modulates the maturation of the immune system, the gut barrier, and the gut–brain axis [47,50]; and (iii) through **neuroendocrine programming** of the hypothalamic–pituitary–adrenal (HPA) axis, which shows persistent alterations in stress reactivity [35].

In a 2024 review, Jovandarić et al. showed that anaerobic fermentation of dietary fiber into short-chain fatty acids (SCFAs) represents a key anti-inflammatory mechanism that shapes the gut microbiota, which is structurally analogous to the process described in Section 5.3 in the context of FASD [51]. The authors emphasize that the early-infant period is a **critical intervention window for correction of dysbiosis** and reduction of chronic disease risk, an observation consistent with the rationale for the intervention protocol proposed in Table 1.

Within the fetal programming framework, FASD may therefore be interpreted as a **developmental disorder with a dual programming profile**: neurodevelopmental (direct ethanol neurotoxicity to the CNS, described in the classical neuroimaging literature) and peripheral–enteric (dysbiosis, SCFA deficiency, barrier damage, and dysregulation of the kynurenine pathway, described in the present review). Each of these programming pathways is a potential target for intervention during infancy, a period of particular susceptibility to modulation because of developmental plasticity. The fetal programming interpretation of FASD extends the intervention perspective beyond classical symptomatic treatment to strategies that modulate the microbiota and gut barrier within a critical neurodevelopmental window.

## 6. How the Gut Generates Depression and Self-Injurious Behavior: The Mechanistic Cascade

The gut–brain axis communicates with the brain through four parallel pathways [42]:

1. The **neural pathway**—via the vagus nerve [52];
2. The **endocrine pathway**—via modulation of the hypothalamic–pituitary–adrenal (HPA) axis;
3. The **immune pathway**—via pro-inflammatory cytokines;
4. The **metabolic pathway**—via microbial metabolites (SCFAs, indoles, and tryptophan metabolites).

In FASD, all four are disrupted simultaneously.

### 6.1. Disrupted Tryptophan Metabolism: From Serotonin to Neurotoxins

Tryptophan is metabolized along three alternative pathways: the serotonin (5-HT) pathway, the kynurenine (KYN) pathway, and the indole pathway [53]. Under physiological conditions, most tryptophan is directed to the serotonin pathway.

In FASD, two mechanisms combine to redirect tryptophan to the kynurenine pathway: **chronic inflammation** (LPS, TNF-α, and IFN-γ) induces the activity of indoleamine 2,3-dioxygenase (IDO), which converts tryptophan into kynurenine, and **the deficit of indole-producing microbiota** weakens the natural negative regulation of the pathway.

The net effect is a **dual hit**: a decrease in the availability of tryptophan for central serotonin synthesis, together with accumulation of neurotoxic metabolites, in particular quinolinic acid, an NMDA-receptor agonist with excitotoxic action on neurons [54].

### 6.2. SCFA Deficiency Destabilizes the Blood–Brain Barrier and Microglia

Erny et al. showed that germ-free or antibiotic-treated mice have an immature, dysfunctional microglial phenotype and that restoring SCFA production by SCFA-producing bacteria reverses this phenotype [39]. In FASD, butyrate deficiency deprives microglia of their anti-inflammatory “brake”. Microglia shift to a persistent pro-inflammatory M1 phenotype, releasing TNF-α and IL-6, performing excessive synaptic pruning, and impairing neuroplasticity in the hippocampus and prefrontal cortex.

### 6.3. HPA-Axis Dysregulation and Stress Hypersensitivity

LPS translocation activates the HPA axis, inducing chronically elevated cortisol concentrations. Cortisol, in turn, further weakens the tight junctions of the intestinal epithelium, closing a self-perpetuating loop: dysbiosis → LPS → inflammation → cortisol → barrier damage → dysbiosis.

### 6.4. Why Classical SSRIs Often Fail

Selective serotonin reuptake inhibitors act by increasing the availability of serotonin at the synapse. If, however, the source problem is a **deficit of serotonin synthesis** (damaged EC cells, tryptophan diverted into the KYN pathway, and dysbiosis), then SSRIs have little “material” to work with. Strategies targeting “gut repair” are **complementary**, not competing, with classical psychiatry.

### 6.5. Alternative and Complementary Mechanisms in FASD Pathophysiology

The focus of the present review on the gut–brain axis is complementary to, not a replacement for, the classical picture of FASD pathophysiology. A fair and balanced discussion of the available evidence requires acknowledgment that the psychiatric phenotype observed in FASD is the result of **multiple parallel mechanisms**, of which the gut–brain axis is one—potentially important, but not the only—element.

**Direct neurotoxicity of ethanol.** The classical interpretation of FASD pathophysiology, well established in the neuroimaging and neuropathology literature, points to a direct action of ethanol on the developing CNS. Jarmasz et al. documented reduced brain volume, disturbances in neuronal migration, disorganization of cortical architecture, corpus callosum hypoplasia, and myelination disturbances in the brains of children with FASD [55]. The neurotoxicity of ethanol includes induction of neuronal apoptosis, impaired proliferation of precursor cells, generation of oxidative stress, and disturbances of trophic signaling. The classical interpretation remains fully empirically justified and constitutes a pillar of the understanding of FASD; the gut–brain axis proposed in the present review is offered as a **complementary mechanism**, accounting for the portion of the phenotype that the classical model does not fully explain (the over-representation of peripheral serotonin disturbances, chronic inflammation, and the influence of the microbiota on microglial development).

**Epigenetic changes.** Ethanol affects the epigenome of the developing fetus through a multi-pathway mechanism involving changes in DNA methylation, histone modifications, and dysregulation of non-coding RNAs. Mews et al. showed that ethanol metabolism directly contributes to acetylation of brain histones, one of the fundamental mechanisms through which prenatal exposure permanently programs the expression of neurodevelopmental genes [49]. Disturbances of one-carbon metabolism (SAM-dependent methylation and the folate–methionine cycle) provide the biochemical rationale for the efficacy of choline supplementation in the RCTs by Wozniak and Jacobson [6,7,20,22]. Epigenetic changes induced by PAE are **potentially reversible** through nutritional interventions that provide methyl-group donors (choline, betaine, folate, and vitamin B_12_).

**Genetic susceptibility of the mother and fetus.** The expression of the FASD phenotype depends not only on the dose and pattern of ethanol exposure but also on genetic variants that modulate alcohol metabolism (alcohol dehydrogenase ADH1B, aldehyde dehydrogenase ALDH2, and cytochrome CYP2E1) both in the mother and in the fetus. Variants with slower metabolic kinetics prolong tissue exposure to ethanol and acetaldehyde. Polymorphisms in genes of the one-carbon pathway (MTHFR, MTR, and MTRR) may modulate the individual response to choline supplementation, an area that warrants further study.

**Environmental and psychosocial modifying factors.** The psychiatric phenotype in FASD is substantially modified by environmental and psychosocial factors. In a cohort of 195 children and adolescents with FASD, Tan et al. reported that the presence of a depressive disorder increased the risk of suicidal behavior fourfold (OR = 4.20), and concurrent involvement with the criminal justice system was an independent predictor of suicidal behavior [5]. The high frequency of Adverse Childhood Experiences (ACEs) in this population creates an additional neurodevelopmental burden independent of the direct mechanism of FASD.

**Co-substance exposures.** The Boston Birth Cohort (Garrison-Desany et al., *n* > 3000 mother–child pairs) showed that prenatal exposure to multiple substances (opioids, tobacco, alcohol, and cannabinoids) has an additive and modifying effect on the risk of ADHD in the offspring, with each additional substance in the maternal history increasing the risk of ADHD by 21% [56]. In the FASD patient population, tobacco co-exposure is particularly common, and a substantial contribution to white-matter pathology in the brain has been demonstrated [57].

**Limitations of translating animal-model findings into human clinical practice.** A substantial share of the mechanistic evidence for the gut–brain axis in FASD, including the role of SCFAs, activation of TLR4 and microglia, dysbiosis with loss of butyrate producers, and increased gut barrier permeability, is derived from rodent models [41,47,48,58,59,60]. The animal model offers control over dose, exposure pattern, and developmental time points; its translation into human clinical practice, however, has important limitations that must be taken into account when interpreting the evidence. First, the alcohol doses and exposure patterns used in rodent models do not fully reflect human social exposure: in humans, PAE is most often episodic (binge) or low-dose sustained and is frequently associated with polysubstance use (Section 9.1). Second, the kinetics of microbiota maturation, immunometabolism, and HPA-axis development differ substantially between rodents and humans: the weaning period in rodents (P21–P22) is not a direct equivalent of the sixth to twelfth month of human life, which complicates the direct transfer of intervention time points. Third, human neuropsychiatric phenotypes (depression, self-injurious behavior, ADHD, and suicidal ideation) do not have fully corresponding behavioral biomarkers in rodents; the animal “anxiety” and “depression” tests measure a narrow aspect of behavior that cannot be treated as an adequate surrogate for human mental health. Consequently, mechanistic data from rodents, however statistically strong, remain at a hypothesis-generating level pending validation in prospective RCTs in humans with FASD.

**Conflicting evidence and the boundaries of current knowledge.** A fair interpretation of the available data requires acknowledgment that not all studies unambiguously support the importance of the gut–brain axis or the proposed interventions in FASD. An RCT of choline supplementation in children with FASD older than 5 years did not show a significant effect on memory, executive function, or attention, suggesting that the therapeutic window may be narrowed to early childhood and that positive results from younger age groups do not extrapolate linearly. The effect observed by Pärtty et al. [18] in healthy infants supplemented with *L. rhamnosus* GG during the first six months of life may be specific to that intervention window and may not carry over to older children or to children with an established FASD phenotype. Meta-analyses of sodium butyrate and psychobiotic supplementation in other pediatric populations (IBS, IBD, ADHD, and ASD) show variable effect sizes, considerable methodological heterogeneity, and a frequent risk of publication bias. Furthermore, part of the proposed mechanism—including the role of quinolinic acid, the exact contribution of LPS, and the clinical significance of the very low TMAO concentrations observed in infants—rests on mechanistic and preclinical evidence and has not yet been verified in prospective clinical studies in children with FASD. A truthful presentation of the current state of knowledge requires acknowledgment of this heterogeneity and avoidance of uniform, “off-the-shelf” therapeutic recommendations. Where this review states a society position, that position is the most recent one available at the time of writing. It should not, however, be read as the only line of evidence because, in three areas, the published literature is not unidirectional, and the reader is entitled to see both sides. First is choline in preterm infants. The current ESPGHAN position does not recommend additional routine supplementation and finds no evidence of benefit in infants fed predominantly breast milk [13]. Against this, randomized data published after that position was drafted show that an enteral supply of 30 mg/kg/day restores plasma choline to concentrations observed in cord blood without serious adverse events and that all four commonly used choline preparations are metabolically safe in this population [15,16]. The society position and the trial data are not in direct contradiction: the former addresses routine supplementation of unselected preterm infants, while the latter addresses attainment of fetal plasma concentrations in a defined clinical situation. The proposal made here belongs to the second question, not the first. Second is lactoferrin. The trial on which the dose in Table 1 is based reported a reduction in late-onset sepsis in very-low-birth-weight neonates [17], and a subsequent meta-analysis of small trials pointed in the same direction. The considerably larger ELFIN trial, however, randomized 2203 very preterm infants to enteral bovine lactoferrin 150 mg/kg/day until 34 weeks of postmenstrual age and found no reduction in late-onset infection (28.9% vs. 30.7%; adjusted relative risk 0.95, 95% CI 0.81–1.10) or in other morbidity or mortality [61]. The current society position, which finds the evidence insufficient for routine use [13], is consistent with the larger trial rather than with the earlier ones. Any use of lactoferrin in the population discussed here therefore rests on a mechanistic rationale and not on a demonstrated clinical effect. Third, the ratio of arachidonic to docosahexaenoic acid. The current preterm position accepts an ARA:DHA ratio between 0.5 and 2 [13], whereas the European Academy of Paediatrics and Child Health Foundation position argues that arachidonic acid should continue to be present in formula, particularly where docosahexaenoic acid is added [9]. This disagreement is unresolved in the primary literature and is presented here as such. In each of these three areas, the protocol proposed in this review departs from, or extends beyond, the current society recommendation. That departure is stated deliberately and openly rather than concealed, and it constitutes a hypothesis to be tested rather than a guideline to be followed.

**Maternal mental health and socioeconomic status.** Women who consume alcohol during pregnancy have a higher frequency of comorbid psychiatric disorders, lower socioeconomic status, and more frequent exposure to domestic violence. Each of these factors independently affects the development of the child through mechanisms of maternal toxic stress, caregiving quality, and the availability of developmental support resources. The model of FASD pathophysiology must therefore account for the fact that the “PAE” effect observed in epidemiological studies encompasses both the direct action of ethanol and the associated background factors.

**Interpretive consequences.** Acknowledging this multiplicity of mechanisms leads to three conclusions. First, the gut–brain axis is a **complementary mechanism** that supplements the classical neurotoxic picture; it does not replace it, and it is not the sole explanation for the FASD phenotype. Second, intervention strategies must be multimodal: alongside interventions targeting the gut–brain axis, classical symptomatic treatment, psychosocial support, prevention of co-exposures, and interventions addressing maternal mental health are all essential. Third, clinical trials of interventions in FASD must control for psychosocial and genetic variables so that conclusions about efficacy are accurately interpreted; the current literature, including RCTs of choline supplementation, largely does not stratify results according to these variables.

## 7. Experimental Evidence Linking the Gut to Behavior

**Vella et al.** [48]: In a rat model of PAE, offspring had reduced butyrate concentrations at the time of weaning, and this reduction was accompanied by an increase in pro-inflammatory cytokines in the amygdala and hypothalamus as early as infancy. These changes persisted into adolescence and correlated with anxiety-like phenotypes [48].

**Busayli et al.** [60]**:** A comprehensive review of studies in rodent models confirms a reproducible trajectory: prenatal alcohol exposure induces microbial dysbiosis, increased gut barrier permeability, and neuroinflammation with activation of TLR4 and microglia, together with reduced luminal SCFA. This pattern is reproducible across different animal models and constitutes a mechanistic foundation for gut–brain axis-targeted interventions in humans [60].

**Shen et al.** [58]**:** In a model of chronic alcohol exposure, administration of butyrate reversed depression-like behaviors, reduced microglial activation, and restored gut barrier integrity [58].

**Lathrop et al. (Brain Behav Immun 2024; research letter): Supplementation with multi-strain probiotics** in pregnant animals modulated the neurodevelopmental trajectory of the offspring [59].

**Pärtty et al.** [18]**:** This study deserves separate emphasis. Seventy-five infants were randomized to supplementation with *Lactobacillus rhamnosus* GG or placebo during the first 6 months of life. At the 13-year follow-up, ADHD or Asperger syndrome had been diagnosed in 17.1% of children in the placebo group and in **0% in the probiotic group** (*p* = 0.008). Children who developed a neuropsychiatric disorder had significantly lower *Bifidobacterium* levels in stool during the first 6 months of life. This is the first prospective study to show that a microbiota-targeted intervention in early life may modulate the risk of neurodevelopmental disorders [18].

## 8. Schematic Overview of the Gut–Brain Axis Cascade in FASD

**Figure 2:**
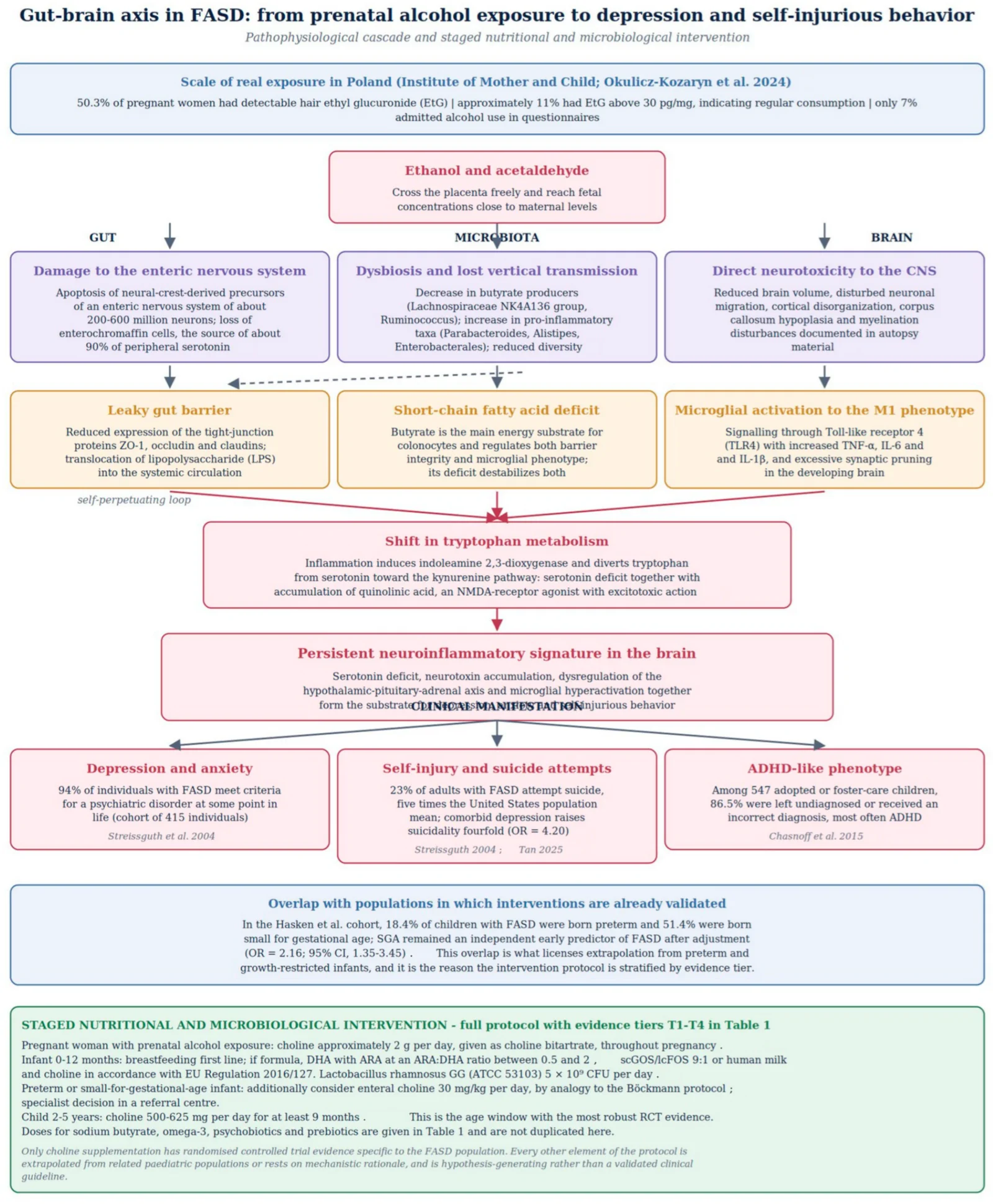
Schematic overview of the gut–brain axis cascade in fetal alcohol spectrum disorder (FASD). The diagram integrates three anatomical compartments–gut, microbiota, and brain–with the four mechanistic pathways described in Section 5, Section 6 and Section 7: (i) damage to the enteric nervous system and enterochromaffin cells with loss of peripheral serotonin production; (ii) alcohol-induced dysbiosis with a decrease in butyrate producers (Lachnospiraceae and Ruminococcus) and an increase in pro-inflammatory taxa (Parabacteroides, Alistipes, and Enterobacterales); (iii) increased permeability of the gut barrier (“leaky gut”) with translocation of lipopolysaccharide (LPS) into the systemic circulation; and (iv) microglial activation to the M1 phenotype via Toll-like receptor 4 (TLR4), together with a shift of tryptophan metabolism toward the kynurenine pathway. The upper panel summarizes Polish epidemiological data [43]. The direct neurotoxicity of ethanol is shown as a parallel, complementary mechanism (right side of the diagram). Abbreviations: PAE–prenatal alcohol exposure; ENS–enteric nervous system; SCFAs–short-chain fatty acids; LPS–lipopolysaccharide; TLR4–Toll-like receptor 4; IDO–indoleamine 2,3-dioxygenase; SGA–small for gestational age; IMiDz–Institute of Mother and Child, Warsaw. Sources cited within the figure: Chasnoff 2015 [3]; Streissguth 2004 [4]; Tan 2025 [5]; Böckmann protocol [15]; enteric nervous system [40]; Okulicz-Kozaryn 2024 [43]; dysbiosis [47]; tryptophan/kynurenine [54]; autopsy material [55]; Wozniak [20,22]; choline in pregnancy [6,7]; LGG dose [18]; ARA:DHA 0.5–2 [13]; Hasken cohort [62].

The cascade described in Section 5, Section 6 and Section 7 is summarized graphically in Figure 2.

## 9. The Real-World Population of Children with FASD: Co-Exposure, Foster Care, Preterm Birth, and Small-for-Gestational-Age Status

The classical mechanistic studies in animals model “pure” PAE: alcohol as the only exposure. Clinical reality is dramatically different. In an autopsy study of 174 individuals with documented prenatal alcohol exposure, Jarmasz et al. showed that **almost all mothers had smoked tobacco**, a substantial proportion had used other substances, and prenatal care had been inadequate or absent [55]. This picture translates into three practical clinical challenges that change the way intervention should be viewed.

### 9.1. Prenatal Co-Exposure: Alcohol Rarely Acts Alone

**Tobacco and nicotine.** Tobacco contains not only nicotine but also tobacco-specific nitrosamines (TSNAs), including NNK (nicotine-derived nitrosamine ketone). In a rat model, Zabala et al. showed that NNK at doses corresponding to the exposure of a smoker induces white-matter damage and myelination disturbances independently of ethanol and that in combined exposure the effect is additive or synergistic [57,63]. Mechanistically, nicotine and NNK cause placental vasoconstriction, fetal hypoxia, oxidative DNA damage, and disturbances of insulin/IGF signaling, effects that partly overlap with those of alcohol and partly act independently. As a result, in a child with PAE + tobacco co-exposure, we observe more pronounced growth restriction, greater myelination deficits, and greater severity of behavioral symptoms than in a child exposed “only” to alcohol.

**Cannabinoids.** Prenatal cannabis exposure disrupts the development of the endocannabinoid system, GABAergic signaling, and cortical circuit organization [64]. The prevalence of cannabis use during pregnancy is increasing globally, and cannabinoids are often used in combination with alcohol and tobacco. In the Garrison-Desany et al. study of more than 3000 mother–child pairs from the Boston Birth Cohort, prenatal opioid exposure was most strongly associated with the risk of ADHD, and the **opioid × cannabis and opioid × alcohol interactions also significantly increased this risk** [56]. This reinforces the argument from Section 4: part of the “ADHD” is in fact a consequence of multi-substance co-exposure.

**Opioids. In a national analysis of United States hospital discharge records covering 2010 to 2017, the rate of neonatal abstinence syndrome rose by 83%, from 4.0 to 7.3 per 1000 birth hospitalizations, while maternal opioid-related diagnoses rose by approximately 130%, from 3.5 to 8.2 per 1000** [65]. **Polysubstance exposure is the rule rather than the exception in this population, with cigarettes and alcohol being the substances most often used alongside opioids; the exact proportions vary between cohorts and are not restated here.** Opioids act through their own mechanism—direct activation of μ receptors on fetal neurons—but they additionally **induce dysbiosis in the mother and offspring, which independently increases pain sensitivity, anxiety, and deficits in emotional regulation** [66]. A child with PAE + opioid exposure therefore has two overlapping dysbioses.

**Practical consequence.** The history of a child with suspected FASD should always include questions about exposure to tobacco, cannabis, and other substances. In an adopted child or a child placed in foster care, where an accurate history is not possible, **probable polytoxicity** should be assumed, and both the clinical assessment and the nutritional intervention should be intensified accordingly. Mechanistically, all these exposures converge at two points: gut dysbiosis and neuroinflammation, which paradoxically reinforces the rationale for interventions targeting the gut–brain axis.

### 9.2. Children in Foster Care: The Lost Vertical Transmission of the Microbiota

The population of children with FASD includes an over-representation of children raised in foster care, adoptive families, or children’s homes. The international data are consistent:

- In the scoping review by Engesether et al. [46], **18.8% of infants and children in foster care met the criteria for FASD**, and among children with FASD **30.5% enter foster care**, almost one in three children with this diagnosis [46].
- In the Colom et al. [67] cohort of children adopted from Russia and Ukraine, **50% met the criteria for FASD** [67].
- In the United States, the prevalence of FASD among children in foster care is 5- to 15-fold higher than in the general population [68].
- Reports from Polish children’s homes include data from individual institutions indicating a concentration of up to 80% of children with a FAS/FASD diagnosis in some settings, consistent with international trends in the highest-risk pediatric populations.

Streissguth showed that 80% of patients with FASD in her cohort were not raised by the biological mother [4]. Additional Polish data from Domin and Mazur show that 53.9% **of children with FAS** have body weight below the 3rd percentile, and 28.8% of the entire FASD population have BMI below the 5th percentile [69], which further complicates nutritional intervention.

**Three real-world clinical constraints that must be acknowledged explicitly:**

1. **Breastfeeding in children’s homes and in foster families is an exceptional situation, not the norm.** A child with FASD placed in foster care usually (a) was not breastfed by the biological mother (alcoholism, polytoxicity, or separation after delivery); (b) does not have access to donor human milk (the Polish Network of Human Milk Banks, although actively developing, prioritizes the distribution of milk to **preterm infants and hospitalized infants**, not to children placed in foster care); (c) is placed in foster care most often only after the period of exclusive breast-milk feeding. The suggestion to “breastfeed a child in foster care” is, in practice, a **rhetorical postulate** rather than a real option. This means that for this population, **the second-line intervention (formula with an optimal composition) is in practice the first-line intervention**.
2. **Instability of care is the rule.** Frequent changes of caregivers and institutions activate the HPA axis, deepen the inflammatory burden, and hamper consistent nutritional intervention. This is a **medical argument for stability of care** to be taken into account in administrative decisions.
3. **The prenatal history is impossible to obtain or unreliable.** Consequently, one should act under a worst-case assumption: PAE + polytoxicity + absence of vertical microbial transmission + instability of care = the maximum burden of adverse factors.

**Absence of breastfeeding: biological consequences.** Breast milk provides *Bifidobacterium* and *Lactobacillus* from the maternal microbiota; human milk oligosaccharides (HMOs) that selectively feed *Bifidobacterium*; lactoferrin, with immunomodulatory and antibacterial properties; and SCFAs, anti-inflammatory cytokines, and secretory IgA. Formula-fed infants show reduced *Bifidobacterium* abundance, increased numbers of antibiotic-resistance genes in the microbiome, and a less mature microbiota profile [70]. In a child with FASD who was additionally not breastfed, there is therefore a **double hit on the microbiota**: prenatally alcohol-induced dysbiosis + inadequate postnatal colonization.

**Early antibiotic therapy.** Children placed in institutional care are more often hospitalized in the first weeks of life (withdrawal symptoms, infections, and low birth weight) and receive antibiotics, further disrupting the early microbiota.

**Practical consequence.** In a child with FASD raised outside the biological family, nutritional and microbiological intervention has a dual justification: it corrects both the deficits arising from PAE and those arising from the lack of breastfeeding. This is the group in which the potential benefit is greatest and for which current clinical guidance is least tailored.

### 9.3. Children with FASD Are Most Often Also Preterm and Small for Gestational Age: Overlapping Populations

The third pillar of the clinical reality of children with FASD is the **over-representation of preterm birth and intrauterine growth restriction (IUGR)**. This observation is central to the intervention strategy: it influences the choice of infant formula (Section 10.10) and, crucially, the rationale for choline supplementation in infants with FASD (Section 10.1).

Hard data:

- In the cohort study by Hasken et al. [62] including 737 randomly selected children assessed at 7 years of age, among 255 children diagnosed with FASD, **18.4% had been born preterm (<37 weeks)**, **51.4% had been born small for gestational age (SGA, <10th percentile)**, and 5.9% met both criteria simultaneously. For comparison, in the non-FASD group, the corresponding proportions were 12%, 28%, and 0.5%. After adjustment for tobacco smoking, maternal age, and education, **SGA remained a strong, independent early predictor of FASD (OR = 2.16; 95% CI, 1.35–3.45)** [62].
- In the meta-analysis by Patra et al., heavy alcohol consumption during pregnancy increased the risk of low birth weight (LBW), preterm birth (PTB), and SGA in a linear, dose-dependent manner [71].
- In the systematic review with meta-analysis by Akison et al. [72], PAE showed an inverse dose–response relationship with birth weight and body length, and the certainty of evidence for SGA and LBW was higher than for other developmental parameters [72].
- In the Polish study by Okulicz-Kozaryn et al., alcohol consumption during pregnancy was associated with a **sixfold higher risk of delivering an SGA infant** [43], replicating the international observations in the Polish population.

In clinical practice, this means that **every second child with FASD is small for gestational age, and nearly one in five is a preterm infant**. A child with FAS born at 33 weeks of gestation with a birth weight of 1800 g is simultaneously the following: 1. a **preterm infant**: a category with published doses of choline, docosahexaenoic acid (DHA), arachidonic acid (ARA), iron, and vitamin D and with a known safety profile of nutritional interventions; 2. a **growth-restricted infant**: a category with published data on choline deficiencies and their metabolic consequences; and 3. a **child with FASD**: a category with published RCTs in children ≥2 years but with an evidence gap for infants.

This overlapping membership in three populations has **crucial importance for the argumentative pathway of the intervention protocol**: the “analogy from the preterm infant population” for a child with FASD is often not extrapolation: it is a statement about the same patient. The diagnostic boundaries here are, in part, arbitrary.

## 10. A Structured Intervention Protocol: Where to Start, What to Supplement, and What to Monitor

**Methodological and regulatory note.** The protocol below is a **clinical proposal grounded in a hierarchy of evidence**, not a formally validated standard of care. **None of the interventions discussed is registered in Poland or the European Union as a treatment for FASD.** Choline, sodium butyrate, GOS/FOS prebiotics (galacto-oligosaccharides and fructo-oligosaccharides), omega-3 fatty acids, *L. rhamnosus* GG, and other strains mentioned are available on the market as dietary supplements, food for special medical purposes (FSMP), or foods for special nutritional use (e.g., infant formulas). Sodium butyrate in microencapsulated form is available in Poland as a food for special medical purposes. Only one intervention—choline—has RCT-level evidence specific to the FASD population. The other proposals are based on RCTs in related pediatric populations (preterm infants, growth-restricted infants, IBD, IBS, ADHD, and ASD), mechanistic studies, and extrapolation. Each intervention should be individualized and monitored, and the entire protocol should be discussed with a specialist in pediatrics, neonatology, pediatric gastroenterology, pediatric neuropsychiatry, and clinical nutrition. Dosing in children under 2 years of age has, in most cases, no formal RCT validation in children with FASD and is presented as an expert proposal based on extrapolation from related infant populations, primarily preterm and growth-restricted infants, who together constitute approximately 64% of the FASD population from birth (Section 9.3).

We propose a **four-stage approach** covering three temporal windows: **prenatal** (mother during pregnancy), **early infancy/preschool** (<2 years), and **preschool/school-age** (≥2 years). Each substance is discussed from the perspective of the active substance and, where relevant, the bacterial strain, without reference to trade names of specific products.

STAGE 1: Baseline assessment (every child);

STAGE 2: Deficit correction and barrier reinforcement (every child with FASD);

STAGE 3: Microbiota modulation (most children with FASD);

STAGE 4: Targeted neuroprotective interventions (selected phenotypes).

**STAGE 1—Baseline assessment**

Before any nutritional intervention, a baseline assessment is required; its results determine the next steps.

**Laboratory tests (mandatory):** Complete blood count with differential; iron status: ferritin, transferrin saturation, CRP (CRP to distinguish inflammatory states that confound ferritin); vitamin D_3_ (25-OH-D); vitamin B_12_ and folate; serum zinc; lipid profile (children with FASD have been reported to show unexpected disturbances of lipid and protein metabolism and deficiencies of fat-soluble vitamins and choline [73]); albumin and total protein; ALT and AST (screening for hepatotoxicity); and TSH (screening for thyroid dysfunction, which often accompanies neurodevelopmental disorders).

**Optional tests (where available):** Fecal calprotectin, an indirect marker of intestinal inflammation; erythrocyte DHA/EPA or the omega-3 index, markers of fatty-acid deficiency; a fatty-acid panel in stool (SCFA), available in selected laboratories; microbiota testing (16S), currently mostly research-based but increasingly available commercially; and hydrogen breath test with lactose or stool lactose concentration, indicated in children with abdominal pain, bloating, and diarrhea that worsen after dairy products (see Section 10.5).

**Extended history, particularly in children in foster care:** Prenatal exposures (alcohol, tobacco, cannabis, and opioids, asked about specifically); feeding during the first year of life (breastfeeding vs. formula, duration, and type of formula); antibiotic use during the first 2 years of life; mode of delivery (cesarean section vs. vaginal delivery); current gastrointestinal symptoms (constipation, diarrhea, abdominal pain, reflux, and bloating after dairy); dietary patterns, food selectivity, and appetite; and gestational age at delivery and birth weight (information critical to dosing of choline and DHA; see Section 10.2 and Section 10.4).

**STAGE 2—Deficit correction and barrier reinforcement**

### 10.1. Choline: Priority Intervention, RCT-Level Evidence (With Three Age-Dependent Pathways)

Choline is the only intervention with RCT-level evidence specific to FASD and—crucially—its efficacy is supported **both in the prenatal and in the postnatal period** but through different mechanisms and target populations. We divide the argument into three pathways: prenatal, early-infancy (<2 years), and postnatal (≥2 years).

#### 10.1.1. Prenatal Pathway (Maternal Supplementation During Pregnancy)

In the RCT by Jacobson et al., choline supplementation in women drinking alcohol during pregnancy attenuated the adverse effects of PAE on growth and early cognitive development of the offspring [6]. In an MRI imaging study, Warton et al. showed that maternal choline supplementation protected the volumes of clinically important brain structures in neonates with PAE—the corpus callosum, thalamus, caudate nucleus, and putamen—and that this correlated with better Fagan test scores at 12 months of age [7]. **Maternal doses in the cited RCTs: Approximately 2 g of choline per day throughout pregnancy, administered as choline bitartrate, is recommended (2 × 2.5 g of choline bitartrate, delivering 2 g of choline cation per day; see the published correction to reference** [6]**).** This pathway is therefore of the greatest importance for **prevention**: in any pregnant woman with documented or strongly suspected alcohol consumption during pregnancy, choline supplementation should be considered, independently of other interventions. Position and evidence for choline in the preterm and infant window: These two statements are often conflated and should be kept apart. The current society position is that additional routine choline supplementation in preterm infants is not recommended and that there is no evidence of benefit in infants fed predominantly breast milk; the same document reaffirms the enteral range of 8–55 mg/kg/day without change and adds that higher intakes appear safe [13]. That is a statement about supplementing unselected preterm infants as a matter of routine. It is not a statement that choline supply is without effect. In the randomized trial by Böckmann et al., an additional enteral supply of 30 mg/kg/day for 10 days restored plasma choline to concentrations observed in cord blood, without serious adverse events, and all four preparations tested were metabolically comparable in this population [15]; the deuterium-labeled work in adults established the contrasting behavior of water-soluble and phospholipid-bound forms in a mature microbiota [16]. In the FASD population specifically, choline supplementation is the only element of this protocol with randomized evidence: 500 mg/day for 9 months in children aged 2.5–5 years, with benefit in non-verbal intelligence, visuospatial function, and working memory sustained at 4-year follow-up [20,22], and approximately 2 g/day antenatally [6,7]. The proposal made in this review therefore addresses a defined clinical situation—documented or strongly suspected prenatal alcohol exposure—and not the routine supplementation of all preterm infants, which is the question the society position answers. The absence of a recommendation to treat everyone is not evidence that no one benefits and neither is it a license to treat everyone.

#### 10.1.2. Postnatal Pathway in Children 2–5 Years of Age

A phase I pilot established that choline at 500 mg/day for 9 months is feasible and well tolerated in children with FASD aged 2.5 to 5 years [21], and the subsequent trial by Wozniak et al. showed preliminary behavioral benefits in the same age range [20]; a 4-year follow-up of the same cohort confirmed a sustained benefit in non-verbal intelligence, visuospatial function, and working memory [22]. Machine learning analysis of blood biomarker profiles in children with FASD, together with an epigallocatechin gallate intervention, illustrates the direction in which biomarker-guided stratification of this population may develop [74]. A cumulative analysis of three RCTs (Wozniak and Warton, total *n* = 104, children aged 2.5–5.9 years) showed that choline supplementation improved elicited imitation memory test scores: the effect was stronger in younger children, suggesting the existence of a **therapeutic window in early childhood**.

**Postnatal dosing (2–5 years):** A total of 500–625 mg/day has been used in clinical trials. For older children, the dose has not been formally established in RCTs; experts suggest 500–1000 mg/day depending on body weight. In 55 children aged 5–10 years, a randomized double-blind trial of 625 mg choline/day as glycerophosphocholine for 6 weeks showed no significant effect on memory, executive function, attention, or hyperactivity [75], suggesting that the **therapeutic window is widest before 5 years of age** and that in older children the intervention is less effective or requires a longer duration.

#### 10.1.3. Choline in Infants with FASD (<2 Years): A Gap in the Guidelines and a Proposal for an Expert Bridge

**RCTs of direct choline supplementation in infants with FASD have not been conducted to date.** This gap does not reflect a lack of mechanistic importance: quite the opposite. Choline is an **essential nutrient** during the first year of life; the requirement in a growing infant (calculated per kilogram of body weight) is the highest across the life course, and during periods of most rapid CNS development, the organism draws on choline more intensively than in any subsequent stage. Omitting this window would therefore be physiologically unjustified.

**Why is the analogy to preterm and growth-restricted infants clinically valid in FASD?** Because these are **to a substantial extent the same children** (Section 9.3): 18.4% of children with FASD are born preterm, and 51.4% are SGA [62]. A child with FAS who is also born preterm or with a low birth weight **simultaneously and medically** meets the criteria of populations in which choline has already been studied, dosed, and considered safe. The argument “no RCT in infants with FASD” loses force when we recognize that a **substantial proportion of children with FASD ARE already in the population in which choline has been studied**. The analogy does not consist of extrapolation “from another disease”: it consists of recognizing that the diagnostic boundaries are, in this case, in part arbitrary.

From this perspective, we consider three infant populations in which choline has been safely administered and from which dosing and safety data can be inherited:

1. **Preterm infants on parenteral and mixed (enteral) nutrition.** In the randomized study by Böckmann et al. [15], preterm infants at 28–32 weeks of gestation received an additional enteral **30 mg/kg/day of choline chloride** for 10 days, which restored plasma choline concentrations to values observed in cord blood; no serious adverse events were reported [15]. The ESPGHAN Committee on Nutrition recommends an enteral choline intake in preterm infants of 8–55 mg/kg/day [76], a range reaffirmed without change in the 2022 position paper, which adds that higher intakes appear safe [13]. It must be stated plainly that the same 2022 position does NOT recommend additional routine choline supplementation in preterm infants and finds no current evidence of benefit in those fed predominantly breast milk [13]; the proposal made here is therefore an extrapolation to the specific case of prenatal alcohol exposure and not an implementation of the society recommendation. Reaching concentrations corresponding to cord blood in practice requires values at the upper end of this range (50–60 mg/kg/day).
2. **Infants with parenteral nutrition-associated liver disease (PNALD/IFALD).** The American Society for Parenteral and Enteral Nutrition (ASPEN) considers choline a critical component in all infants requiring TPN, citing the association of choline deficiency with progressive fatty liver disease [51 review].
3. **Term infants with choline deficiency before adoption.** In the study by Fuglestad et al., choline concentrations at the time of adoption in post-institutional children predicted cognitive and motor development in subsequent months, suggesting an important role for choline in a group that substantially overlaps with the FASD population.

**Proposal of an expert recommendation for infants with FASD (<24 months of age):**

- **First line (physiologically preferred):** breastfeeding—human milk contains choline in the form of phosphatidylcholine and sphingomyelin at approximately 150 mg/L; supplementation of the breastfeeding mother (continuation of the maternal dose from pregnancy) raises the choline concentration in milk.
- **Second line (when breastfeeding is not available, the most common situation in foster care):** infant formula with a choline content in accordance with current EU regulations (the regulated unit is mass per 100 kcal, not per 100 mL: the EFSA minimum for infant formula is 25 mg choline/100 kcal, and the ESPGHAN range for preterm infants is 7–50 mg/100 kcal [13,14,76]).
- **Third line, in selected clinical situations (to be considered by a specialist):** additional choline supplementation in an infant with FASD in the setting of documented polytoxicity, preterm birth, low birth weight, or placement in foster care without access to maternal milk, by analogy to doses safely used in preterm infants (30 mg/kg/day enterally; this is an **expert proposal, not a registered indication**, and requires individual consultation in a referral center).

**Position of the authors:** The absence of RCTs in infants with FASD is a knowledge gap, not an argument against intervention. The mechanistic, population, and pharmacokinetic bases for considering choline in the first 24 months of life in a child with FASD are strong, particularly in a child who is simultaneously preterm or growth-restricted. This article proposes that **the initiation of prospective RCTs in infants with FASD is a research priority for the next decade**; until such studies are published, individualized expert practice based on the analogy to populations with an established safety profile is warranted.

**Choline form and a note on TMAO in the infant population.** In the adult literature, the issue of trimethylamine-N-oxide (TMAO)—a metabolite generated from the breakdown of choline by the gut microbiota (choline → trimethylamine → hepatic oxidation to TMAO)—has been widely discussed; high concentrations of TMAO in adults are associated with a pro-inflammatory, prothrombotic, and atherogenic profile [60,77,78]. Water-soluble forms (choline chloride and choline bitartrate) generate the most TMAO in adults; alpha-glycerophosphocholine (alpha-GPC) generates intermediate amounts; and the lipid forms naturally present in human milk—phosphatidylcholine (PC, including POPC), phosphorylcholine (PCho), and sphingomyelin (SPH)—generate the least [60,74,78].

The situation in the target population of the present review—**preterm infants and infants during the first 24 months of life**—differs, however, in a substantive way. In a randomized clinical trial by Böckmann et al. [15] including 32 preterm infants (28 + 0 to 32 + 0 weeks of gestation), four choline preparations (chloride, bitartrate, alpha-GPC, and egg-PC) were compared at a dose of 30 mg/kg/day for 48 h. **All four preparations produced comparable increases in plasma choline concentrations without significant differences between groups** (*p* = 0.2 at baseline; all preparations *p* < 0.05 vs. baseline at 51–54 h), with TMAO remaining at very low concentrations (0.03–0.08 µmol/L, without significant differences between groups) [15]. This result contrasts with an earlier study by the same group performed in healthy men (Böckmann et al. *Eur J Nutr* 2023, D9-labeled model), which showed that in adults with a mature microbiota, the D9-POPC (phosphatidylcholine) preparation generated significantly lower D9-TMAO concentrations than water-soluble forms (D9-choline chloride, D9-phosphorylcholine, and D9-GPC) [16]. In adults with normal renal function, choline supplements, unlike eggs, raised fasting TMAO concentrations [79], whereas dietary phosphatidylcholine did not [77]. Taken together, these studies confirm that the capacity to convert choline to TMA and then to TMAO **depends on the maturity of the gut microbiota**: in preterm infants and young infants, this capacity is undeveloped, and consequently **choline supplementation—regardless of the preparation used—does not carry the prothrombotic or cardiovascular risk typical of adults** [15,16].

In this context, the preference for particular preparations in the present protocol rests on three arguments that are independent of TMAO:

1. **Physiological argument.** The PC, alpha-GPC, phosphorylcholine, and sphingomyelin forms are natural components of human milk and constitute the physiological substrate of choline in the neonatal and infant periods. Reconstructing this profile in an infant formula is consistent with the goal of reproducing the conditions of natural feeding, an overriding goal in a population deprived of biological maternal milk.
2. **Kinetic argument.** In the Böckmann et al. study, alpha-GPC produced **the most rapid increase in plasma choline in preterm infants** (a significant rise was visible as early as 6 h after the start of supplementation, *p* = 0.01) [15]. In a clinical scenario requiring rapid correction of the deficit, a preparation with faster kinetics is preferred.
3. **Long-term preventive argument.** Choline supplementation in FASD is not confined to infancy; RCT-validated doses in children aged 2.5–5 years are 500 mg/day (the Wozniak protocol) [20,22]. As the gut microbiota matures, the capacity to generate TMAO increases. Choosing the PC/egg-PC/phosphorylcholine form in infancy allows the same strategy to be safely continued into the preschool and school-age periods without the need to change the preparation.

**Choline bitartrate and choline chloride preparations**—most often used in the key FASD RCTs (Wozniak 2015, 2020 [20,22]; Jacobson 2018 [6])—remain **fully safe in the preterm and infant population** [15], and their clinical validation in this indication is the strongest. Their use in a clinical scenario in which the preferred preparations are unavailable or not economically accessible is substantively justified. In infant formulas, choline occurs mainly as free choline and as milk- or soy-derived phosphatidylcholine—forms that are naturally TMAO-neutral—so the discussion of TMAO-generating preparations concerns mainly supplements added to feeding, not the formula itself [37].

Citicoline is another alternative used in adult cardiology and neurology, but the absence of RCTs in the pediatric FASD population precludes its recommendation in this indication.

**Mechanism of action:** Choline is a methyl-group donor for DNA methylation. The mechanistic rationale is reinforced by the work of Mews et al., which showed that acetate derived from alcohol metabolism is directly incorporated into brain histones, leaving a persistent “epigenetic footprint” of exposure [49]; choline supplementation partially counteracts this mechanism. Choline is also a precursor of phosphatidylcholine (stabilization of neuronal membranes) and of acetylcholine (attention function).

**Adverse effects:** These are rare; they include an unpleasant “fishy” odor of urine and body at high doses (a harmless symptom) and occasional nausea.

### 10.2. Correction of Micronutrient Deficiencies

**Iron**: This is individualized on the basis of the panel (Hb, ferritin, transferrin saturation, and CRP). A child with PAE may have iron deficiency independently of the mother’s iron status [12]. Well-tolerated compounds are preferred (ferrous sulfate at a dose adjusted for age/weight; alternatively iron polymaltose). **Avoid excessive supplementation**: iron aggravates oxidative stress.

**Vitamin D_3_: At 25-OH-D <30 ng/mL, cholecalciferol is recommended in accordance with the current Polish guideline: 600–1000 IU/day in children aged 4–10 years and 1000–2000 IU/day in adolescents, adjusted for body weight and dietary intake and corrected to reach the target concentration** [80]. **Zinc: At confirmed deficiency, 5–10 mg/day of elemental zinc is recommended. This figure is expert opinion: it sits between the age-dependent recommended intakes for children and the repletion regimens of 0.5–1 mg/kg/day used in the deficiency literature, and no guideline states it as such for this population.**

**Vitamin B_12_ and folate**: Supplementation is recommended at documented deficiency; in children with FASD, paradoxically elevated concentrations of B_12_ have been reported [81], so routine supplementation without laboratory assessment is not indicated.

### 10.3. Omega-3 Fatty Acids (DHA + EPA) and the Balance with ARA

The previously introduced “500–1000 mg DHA + EPA in children” requires refinement: in children with FASD, the typical dose used in the general population cannot be prescribed without consideration of the developmental context. Four principles must apply simultaneously here:

1. **In the prenatal and early-infancy period, the priority is DHA, not EPA.** DHA is the main structural fatty acid of brain and retinal phospholipids: it plays a “building” role, whereas EPA plays a mainly immunomodulatory role, and its conversion to DHA in the infant organism is very limited (<5%). In addition, in animal models of PAE, DHA supplementation restored glutathione concentrations and protected against ethanol-induced oxidative damage [82]. High doses of EPA in an infant may **displace arachidonic acid (ARA) from membrane phospholipids** through competition for desaturases and acyltransferases, which is mechanistically undesirable.
2. **Arachidonic acid (ARA, omega-6) is NOT “the bad omega-6”.** The widely used rhetoric that “omega-6 fatty acids are not recommended” refers to the pro-inflammatory linoleic acid (LA) line from vegetable oils, not to long-chain ARA. ARA is **essential** for the infant in a proportion parallel to DHA. In human milk, ARA averages ~0.5–0.7% of fatty acids and DHA ~0.2–0.4%; the ARA:DHA ratio in human milk is therefore ~1:1 to 2:1. The current ESPGHAN position for preterm infants is broader, recommending DHA 30–65 mg/kg/day and ARA 30–100 mg/kg/day with an ARA:DHA ratio between 0.5 and 2, which supersedes the narrower 2010 ranges and no longer excludes formulations in which DHA exceeds ARA [13].
3. **Age-based recommendations** [8]**:** These are as follows: infants 0–6 months: AI 100 mg DHA and 140 mg ARA/day; infants 6–24 months: 100 mg DHA/day, with ARA in an equivalent amount; children 2–4 years: 100–150 mg DHA + EPA/day; children 6–10 years: 200–250 mg DHA + EPA/day; and children ≥ 11 years: 250–500 mg DHA + EPA/day.
4. **In infant formulas in the EU, since 2020, DHA is mandatory (minimum 20 mg/100 kcal)**, but the requirement to add ARA has been withdrawn; this is subject to ongoing debate in the pediatric community (the position of the European Academy of Paediatrics and Child Health Foundation [9]: ARA should still be present in formulas, particularly when DHA is added).

**Proposals for different age windows in a child with FASD:**

**Pregnancy and breastfeeding (mother).** This presents the strongest data. In accordance with the EFSA and Polish society consensus: **200 mg DHA/day for the pregnant and breastfeeding woman** as an absolute minimum; in high-risk situations (PAE, polytoxicity), **at least 300 mg DHA/day is recommended**, with a preference for fish or algal oil with a documented composition. EPA at doses parallel to DHA in the breastfeeding woman is acceptable.

**Infant 0–12 months, breastfed.** Here, there is no need for direct infant supplementation: DHA is provided by the mother. If needed (mother on a vegan diet, with low intake of marine fish), a dose of 100 mg DHA/day for the infant in drop form is recommended, **preferentially DHA alone from microalgae or in a DHA:EPA ratio of at least 4:1, ideally 10:1** (i.e., avoid fish oil preparations intended for adults, in which the ratio is sometimes reversed, with EPA dominating over DHA).

**Infant 0–12 months, not breastfed (the most common situation in foster care in a child with FASD).** Infant formula as the primary source: choose preparations with DHA content in line with EU recommendations (≥20 mg/100 kcal, optimally 0.3–0.5% of fatty acids) **and with ARA added in an ARA:DHA ratio of at least 1:1**, in accordance with the EAP/CHF position [9]. If the chosen formula does not contain ARA, additional supplementation with DHA alone is **not indicated** because it may deepen the imbalance; a change of formula is preferable. **We do not give infants adult fish oil** (a combination of DHA + high EPA without ARA is not physiological for an infant).

**Preterm infants with FASD. The current ESPGHAN position recommends DHA 30–65 mg/kg/day together with ARA 30–100 mg/kg/day, at an ARA:DHA ratio between 0.5 and 2** [13]; **the narrower 2010 ranges of 12–30 mg DHA/kg/day and 18–42 mg ARA/kg/day** [76] **are superseded.** Supplementation should be part of the nutritional protocol of the neonatal unit.

**Child 1–2 years.** Continuation of DHA 100 mg/day from additional dietary sources (fatty marine fish 1–2× weekly: salmon, mackerel, sardines, and herring, in safe portions of 50–100 g, with a preference for species with a low risk of methylmercury) or a preparation of 100 mg DHA + 100 mg ARA (in accordance with the study by Lien et al. showing a neurodevelopmental benefit of this combination in children 12–24 months [25]) is recommended.

**Child 2–6 years with FASD. EFSA did not set an adequate intake of EPA plus DHA for children aged 2 to 18 years, judging the data insufficient, and advises instead that dietary guidance for children follow that for adults, namely one to two fatty fish meals per week or approximately 250 mg of EPA plus DHA per day** [83]. **The range proposed here, 150–500 mg of DHA plus EPA per day with a DHA-dominant ratio (DHA:EPA at least 2:1, preferably 3:1 or higher), brackets that figure; the DHA-dominant ratio is expert opinion, since no source specifies a ratio for this age group.** Higher doses may be used in the setting of a documented DHA deficit in erythrocytes.

**Child ≥ 6 years with FASD.** The suggested dose is 250–1000 mg DHA + EPA/day; here, the ratio may be more balanced (DHA:EPA 1:1 to 2:1) because the child is no longer in a period of exponential CNS growth, and EPA becomes clinically useful for its anti-inflammatory effects. **In a child with predominantly depressive symptoms**, data from the adult literature indicate that preparations with **EPA ≥ 60% of the DHA + EPA total** have a stronger antidepressant effect than DHA-dominant preparations, which is worth taking into account in the choice.

**Form.** Fish oil in the triglyceride (TG) form is better absorbed than in the ethyl-ester (EE) form. In children with FASD with documented disorders of fat digestion and absorption (frequent in this population due to low BMI and deficiencies of fat-soluble vitamins [73]), the **TG form is preferred**. Vegan/infant alternative: Microalgal oil (*Schizochytrium* sp., *Crypthecodinium cohnii*) can serve as a source of pure DHA without EPA, ideal for the youngest infants. Preparations with a certificate of contaminant control (heavy metals, dioxins) are preferred.

**Three principles that distinguish our position from the general pediatric one: In an infant, we deliver DHA, not the DHA + EPA combination**: high EPA in an infant is not physiological; **ARA must accompany DHA** in the first 24 months. This is not “an omega-6 to avoid” but an essential structural fatty acid. **In an older child with predominant depression, we choose an EPA-dominant preparation**, the reverse of the choice in the younger child.

### 10.4. Reinforcement of the Gut Barrier: Sodium Butyrate

Butyrate is the central metabolite discussed in Section 5, Section 6 and Section 7. Butyrate deficiency in children with PAE is one of the better-documented pathophysiological phenomena [48]. Supplementation with exogenous sodium butyrate is a rational first-line intervention to repair the gut barrier in children after 2 years of age.

**Evidence in children:** In a randomized placebo-controlled trial in 88 children and adolescents aged 7–18 years with newly diagnosed IBD, microencapsulated sodium butyrate 150 mg once daily for 12 weeks induced remission in 81.8% of patients vs. 47.7% on placebo, with significantly lower CRP and a greater fall in fecal calprotectin [84]. In an RCT in children with IBS (51 patients), calcium butyrate 500 mg/day for 8 weeks achieved treatment success in 73% of patients vs. 3.8% in placebo; metagenomic analysis showed increases in *Lachnospiraceae* and decreases in *Ruminococcus gnavus* [23].

**Dosing in children ≥ 2 years: It is most commonly 150–300 mg twice daily, in a microencapsulated form released in the small intestine and colon, in line with the dosage review by Banasiewicz et al.** [85]. Standard dose units available in Poland as a food for special medical purposes contain 150 mg per capsule.

**Age below 2 years. Sodium butyrate has not been tested in RCTs in infants, and most preparations in hard-capsule form are not suitable for administration.** In this age window, the strategy should be **indirect, based on endogenous butyrate production from prebiotic fermentation** (see Section 10.5: GOS/FOS and HMOs) and on rebuilding the population of butyrate-producing bacteria through targeted probiotics. Direct sodium butyrate supplementation in infants should be considered only under specialist conditions, on an individualized basis, and outside standard practice.

**Mechanism:** Butyrate increases the expression of ZO-1, occludin, and claudins, stimulates MUC2 mucin production, induces sIgA, inhibits NF-κB, and—after crossing the blood–brain barrier—inhibits histone deacetylases in the brain and shifts microglia from the M1 to the M2 phenotype [24,29].

**Duration:** At least 8–12 weeks is necessary to allow for assessment of clinical effect.

**Adverse effects:** These are rare and include an unpleasant (sulfurous) odor and bloating in the first days.

**Note on extrapolation. Position and evidence: The pediatric randomized evidence for oral butyrate is not unidirectional, and both directions are set out here. In children with irritable bowel syndrome, calcium butyrate supplementation reduced abdominal pain and overall symptom severity, with treatment success in 73% vs. 3.8% of controls, alongside a shift towards short-chain-fatty-acid-producing taxa** [23]. **In a multicenter placebo-controlled trial in 72 children and adolescents aged 6–18 years with newly diagnosed inflammatory bowel disease, however, sodium butyrate 150 mg twice daily added to standard therapy for 12 weeks produced no difference in the remission rate or in median disease activity (*p* = 0.37 and *p* = 0.31), with no adverse events reported** [86]. **That trial used the lower of the two doses proposed in** Table 1**, so the negative result cannot be dismissed as underdosing. The contradiction is sharper still: a second randomized trial in the same indication, in 88 newly diagnosed children aged 7–18 years, used HALF that daily amount—150 mg once daily rather than twice—and reported remission in 81.8% vs. 47.7%** [84]. **Two randomized trials in newly diagnosed pediatric inflammatory bowel disease, both twelve weeks long, therefore point in opposite directions, and the lower-dose trial is the positive one, which excludes a simple dose–response explanation. What differs between them is the preparation and the release profile: unprotected butyrate given orally is largely absorbed in the proximal small bowel, so colonic delivery depends on pH-sensitive or double-layer encapsulation rather than on the nominal dose** [86]. **Any proposal made here therefore concerns microencapsulated preparations specifically and rests on a mechanistic premise that has not been tested in FASD. The RCT evidence for sodium butyrate is derived from pediatric IBS and IBD populations, not from the FASD population.** The rationale for use in children with FASD rests on the pathophysiological premise (butyrate deficit in FASD, documented in animal models) and on the general safety profile of the preparation; no RCTs verifying the behavioral efficacy of sodium butyrate in the FASD population have been conducted to date. The recommendation therefore remains at T2 (extrapolation) and warrants validation.

**STAGE 3—Microbiota modulation**

### 10.5. GOS/FOS Prebiotics: First Line in Infants and Young Children

In a child with FASD who is not breastfed or after antibiotic therapy, GOS/FOS prebiotics are mechanistically first line. The **scGOS/lcFOS 9:1** mixture mimics the composition of human milk oligosaccharides.

**Evidence:** Intake results in increases in *Bifidobacterium* and *Lactobacillus*, increased SCFA production, and lower luminal pH [27,36]; reduced frequency of infections and allergies during the first 2 years of life [28]; and reduced constipation and improved stool consistency [37].

**Dosing in infants:** These are most often delivered as a component of an infant formula (starter and follow-on formulas fortified with scGOS/lcFOS or HMOs; see Section 10.10). In older children, oligosaccharide preparations at a dose of 1–2 g GOS/FOS/day are suggested; this figure is expert opinion, since none of the trials cited above extends beyond infancy, and no source states a gram-per-day dose for children past the first year. Duration: long-term (months to years). Position and evidence: The ESPGHAN Committee on Nutrition concluded that the data then available did not permit recommending the routine use of prebiotics or probiotics as food supplements in preterm infants and that efficacy and safety should be established for each product [76]. The two randomized trials in preterm infants on which that conclusion rested used formula concentrations of 8 and 9 g of scGOS/lcFOS per liter and reported increased fecal bifidobacteria, lower stool pH, and faster gastrointestinal transit, without data on necrotizing enterocolitis or long-term outcomes [76]. The trials supporting the present section were conducted in term formula-fed infants [27,28,36,37]. The recommendation made here therefore rests on infant data and on mechanistic reasoning, and neither the pediatric dose nor the long-term outcome has been established.

### 10.6. First-Line Probiotic: Lactobacillus rhamnosus GG (ATCC 53103)

Of all available probiotic strains, *L. rhamnosus* GG (ATCC 53103) has the largest evidence base in children (>200 RCTs); a safety profile confirmed from the neonatal period onward, noting that the current ESPGHAN position on probiotics in preterm infants is strain-specific and does not list *L. rhamnosus* GG among the strains with demonstrated efficacy for necrotizing enterocolitis or mortality, so its use here rests on the pediatric rather than the neonatal evidence base [19]; **a prospective RCT showing a reduction in the risk of ADHD and autism spectrum disorders** in children supplemented during the first 6 months of life [18]; an RCT in a pediatric ADHD population showing improvement in quality of life (PedsQL) [87]; and mechanistically demonstrated stabilization of the gut barrier (tight junctions, mucin, and sIgA).

**Dosing:** A total of 10^9^–10^10^ CFU/day (1–10 billion units) is recommended; in infants, 5 × 10^9^ CFU; and in older children, 1 × 10^10^ CFU.

**Form:** Suspension/drops are best for infants and young children; capsules are recommended for older children. In Poland, *L. rhamnosus* GG is available in several product lines as a dietary supplement or a food for special medical purposes; the selection should be guided by **the strain identifier (ATCC 53103)** rather than by trade name.

**Duration:** Their use should last at least 3 months, optimally 6–12 months.

**Note on extrapolation.** The evidence from Pärtty et al. [18] is derived from a population of healthy infants (not FASD), with supplementation delivered during the first 6 months of life. Extrapolation to a child with FASD—particularly to an older child—rests on a mechanistic premise rather than on a direct FASD-specific RCT. The effect observed in the early-infancy window may be specific to that period and may not transfer linearly to an older child with an already-established phenotype. Level of evidence: T2.

### 10.7. Psychobiotics: When Depressive and Anxiety Symptoms Predominate

“Psychobiotics” are probiotics with documented effects on CNS function. The best-studied strains are as follows:

- ***Lactobacillus plantarum* PS128**—in an RCT in 80 boys with ASD (aged 7–15 years), 4 weeks of supplementation improved oppositional–defiant behavior and anxiety [30]; mechanistically, the strain influences dopamine and serotonin production;
- ***Lactobacillus helveticus* R0052 + *Bifidobacterium longum* R0175**—in meta-analyses in adults, reductions in anxiety and depressive symptoms [88];
- ***Bifidobacterium longum* 1714**—an RCT in healthy adults with an effect on stress perception and memory;
- ***Bifidobacterium infantis* 35624**—mechanistic evidence for modulation of tryptophan and the HPA axis.

**Indications in FASD:** This presents as a child with predominant anxiety–depressive symptoms, not necessarily meeting the full criteria for depression.

**Dosing: For *L. plantarum* PS128, the randomized trial in children used capsules containing 3 × 10^10^ CFU taken twice daily, that is 6 × 10^10^ CFU/day** [30]; **other strains should follow their own documented schedules. Duration: The trial in children lasted 4 weeks** [30]; **schedules of 8 to 12 weeks are used with other psychobiotic strains but have not been tested for PS128 in this population.**

**Important:** Psychobiotics **do not replace psychotherapy or—in severe depression—pharmacotherapy**. These are complementary interventions.

**Note on extrapolation. Position and evidence: The pediatric meta-analytic evidence for psychobiotics is negative and must be stated as such. A systematic review and meta-analysis of five randomized trials in 692 children and adolescents (mean age 7.33 years) found no improvement in depressive symptoms with probiotics (standardized mean difference 0.04; 95% CI, −0.33 to 0.41; *p* = 0.84), and none in the subgroup with neurodevelopmental diagnoses (standardized mean difference −0.11; 95% CI, −0.73 to 0.51; *p* = 0.72); heterogeneity was high, and the certainty of evidence was graded very low** [89]. **Notably, one of the included trials tested the same strain proposed here, *L. plantarum* PS128, in children with Tourette syndrome and did not show an effect on depressive symptoms. The authors of that meta-analysis are explicit that their result cannot rule out a therapeutic effect because no included trial recruited children with a diagnosis of depression or anxiety and symptom severity at baseline was low. The position taken in this review is therefore the narrower one: the trial evidence does not support psychobiotics for depressive symptoms in unselected children, and their consideration in FASD is confined to a defined phenotype, as a hypothesis. All RCT evidence for psychobiotics is derived from adult populations (depression or anxiety) or children with ASD, not from the FASD population.** The rationale for use in FASD rests on a mechanistic premise (shared serotonergic and tryptophan pathways) and on the safety profile of these strains. No RCTs in the FASD population have been conducted; level of evidence: T3 (mechanistic + extrapolation from related populations).

### 10.8. Synbiotics in the ADHD-Like Phenotype

In an RCT in children with ADHD, a synbiotic composed of four strains (*Pediococcus pentosaceus* 5-33:3, *Lactobacillus casei* ssp. *paracasei* 19, *Lactobacillus plantarum* 2362, and *Leuconostoc mesenteroides* 32-77:1) together with four plant fibers (β-glucans, inulin, pectin, and resistant starch) did not produce a definite overall effect on core ADHD symptoms; improvements were confined to prespecified subgroups with elevated baseline vascular inflammation (soluble VCAM-1), in whom autistic symptoms in children and emotion regulation in adults improved [26]. Such a combination may be considered in a child with FASD who has predominant ADHD-like symptoms.

**Note on extrapolation. Position and evidence: The pediatric meta-analytic evidence for probiotics in ADHD is likewise negative. A meta-analysis of seven randomized trials in 379 children and adolescents (mean age 10.37 years) found no significant improvement in total ADHD symptoms (standardi\zsed mean difference 0.25; *p* = 0.12), in inattention (0.14; *p* = 0.3) or in hyperactivity and impulsivity (0.08; *p* = 0.54) and concluded that current evidence shows no difference in efficacy between probiotics and placebo for ADHD symptoms** [90]. **Subgroup differences favoring multi-strain over single-strain regimens and adjunctive over standalone use did not reach significance, but they are the reason a multi-strain synbiotic rather than a single strain is discussed here. Taken together with the absence of a definite overall effect in the trial cited above** [26], **the proposal in this section should be read as a mechanistic hypothesis about a specific phenotype and not as an evidence-based recommendation for ADHD-like symptoms. The RCT evidence for the above combination is derived from children with ADHD without FASD.** The rationale for use in the FASD population rests on the high frequency of co-occurring ADHD-like symptoms and shared pathophysiological mechanisms but has not been verified in an FASD-specific RCT. Level of evidence: T3.

**What NOT to use:**

- **Probiotics with unclear strain identification**: Products without a precise strain identifier (e.g., “*Lactobacillus acidophilus*” without a collection identifier such as ATCC or DSM) have limited clinical value; in clinical microbiology, **strain ≠ genus ≠ species**.
- ***Saccharomyces boulardii*** in a child with a central catheter or immunosuppression: risk of fungemia.
- **Multi-strain “the more the better” preparations** without specific evidence for the given combination: *L. rhamnosus* GG (ATCC 53103) acts differently from *L. rhamnosus* GR-1 or LR32. The number of strains in a preparation does not correlate with efficacy.
- **The multi-strain eight-bacterial preparation at a high concentration (450 billion CFU)** used in pouchitis and hepatic encephalopathy: **no RCTs in the pediatric FASD population**; reserved for specialist indications.

### 10.9. Lactase and Secondary Lactose Intolerance: A Symptomatic, Not a Causal, Intervention

Abdominal pain, bloating, and diarrhea worsening after dairy products are common symptoms in children with FASD, particularly in those with documented dysbiosis and features of intestinal inflammation. The clinical question of whether to administer the enzyme lactase “on the spot” requires distinguishing the mechanism.

**Lactase deficiency in children with FASD is usually secondary, not primary.** Primary lactase deficiency (congenital alactasia) is extremely rare (~40 documented cases worldwide). Adult-type hypolactasia appears genetically in the population from ~5 years of age. In contrast, **secondary lactase deficiency**—resulting from damage to the intestinal epithelium—is common and well documented in acute infectious enteritis, IBD, celiac disease, SIBO, and mucosal inflammatory conditions. Mechanistically, this is a consequence of **loss of lactase from the tips of the villi**: as a brush-border enterocyte enzyme, lactase is the most susceptible to loss when the epithelium is damaged.

In a child with FASD with dysbiosis and a leaky gut barrier, this mechanism is **probable and common**, but it is a **symptom rather than a cause** of the problem. Exogenous lactase (as enzymatic replacement therapy in drops or tablets) will attenuate the gastrointestinal symptoms but **does not repair the source mechanism: epithelial damage, dysbiosis, and SCFA deficiency**.

**Practical position.** Routine administration of lactase to every child with FASD who has abdominal pain is not mechanistically justified and carries the risk of two clinical errors: 1. **Omission of diagnostic work-up**: Abdominal pain in a child with FASD may result from constipation, SIBO, IBS-D, IgE- or non-IgE-mediated food allergy, reflux, or other causes requiring targeted treatment. 2. **Failure to institute causal interventions**: These include barrier reinforcement (sodium butyrate from 2 years, prebiotics, and *L. rhamnosus* GG), microbiota modulation, and correction of deficiencies.

**When lactase is justified.** Enzymatic lactase is reasonable **as a symptomatic bridge** in the following settings: documented secondary lactose intolerance (lactose hydrogen breath test or stool lactose concentration); a child with markedly impaired quality of life due to symptoms after dairy, during the period of implementing a causal intervention; or the convalescent period following acute enteritis with osmotic diarrhea.

**What is preferred before enzyme use.** In a formula-fed infant with abdominal pain and bloating, **before reaching for a lactose-free product or exogenous lactase**, it is worth considering a formula with **partially hydrolyzed protein (pHF) and reduced lactose content** (in many studies, this attenuates symptoms of formula intolerance without eliminating lactose as a substrate for bifidobacteria); a formula with **GOS/FOS or HMOs** supporting the growth of bifidobacteria; and **elimination of lactose not as the first line** (lactose is a natural substrate for *Bifidobacterium*, and its elimination in an infant weakens bifidobacterial colonization).

In older children, the introduction of fermented milk products (yogurt and plain kefir) in which part of the lactose has already been broken down by lactic acid bacteria is beneficial.

### 10.10. Choice of Infant Formula in a Child with FASD Who Is Not Breastfed

If breastfeeding is not possible (the most common situation in foster-care children), the choice of formula becomes an important clinical decision. **No formulas dedicated to FASD exist**, but preparations can be selected according to a composition supportive of the gut–brain axis.

**What to prefer in the first-choice formula:** A **formula based on whole cow’s milk protein** is the standard in a healthy infant without allergy. **There is no basis for routine selection of hypoallergenic formulas** (extensively hydrolyzed formula [eHF]) in a child with FASD without documented cow’s milk protein allergy (CMPA). **Added GOS/FOS or HMOs** (e.g., 2′-FL, increasingly available in formulas since 2018) is preferred. **Full lactose content** (do not reduce routinely) is recommended, as lactose is a natural substrate for *Bifidobacterium* growth. **DHA at a level in accordance with current EU regulations** is important (mandatory since 2020 in starter formulas, minimum 20 mg/100 kcal). **Added ARA in an ARA:DHA ratio of at least 1:1**, in accordance with the EAP/CHF position [9] is crucial in a child with FASD. **Choline** in the range in accordance with ESPGHAN recommendations is preferred. **Adequate content of iron, zinc, vitamin D, and iodine is important.**

**What to avoid in a child with FASD without clinical indications: Lactose-free formulas**: In the absence of documented secondary lactose intolerance, this represents unnecessary elimination of a beneficial prebiotic substrate. **Soy formulas**: According to ESPGHAN, these are not recommended in healthy term infants under 6 months; 8–14% of infants with CMPA also react to soy. **Extensively hydrolyzed formulas (eHFs) and amino-acid formulas (AAFs) used prophylactically**: These are reserved for clinically confirmed CMPA. **Goat and other mammalian milk formulas** are not a safe alternative in CMPA due to antigen cross-reactivity.

**Situations requiring special preparations: Confirmed CMPA**: eHF formula is recommended as the first line and AAF where eHF is insufficient (~10% of children). **Preterm birth (FASD often coexists with preterm birth—18.4%** [62]**)**: A preterm-infant formula is necessary, continued in accordance with the neonatal protocol; after discharge, continuation with a post-discharge preterm formula in accordance with ESPGHAN is advised. **Small-for-gestational-age status (FASD often coexists with SGA—51.4%** [62]**)**: This necessitates a high-energy formula enriched with protein, DHA + ARA, choline, and micronutrients, to be individualized by a neonatal dietitian. **Marked bloating and colic**: It is important to consider a formula with partially hydrolyzed protein (pHF) and reduced lactose content as a 2–4-week trial.

**STAGE 4—Targeted neuroprotective interventions (selected phenotypes)**

### 10.11. N-Acetylcysteine (NAC): Antioxidant Support

NAC is a precursor of glutathione, the main endogenous antioxidant, deficiency of which is one of the mechanisms of acetaldehyde toxicity. In animal models of PAE, NAC reduces oxidative stress and improves behavioral outcomes. In the pediatric population, NAC has had a safety profile established for decades (as a mucolytic and as an antidote to paracetamol). Doses of 600–2700 mg/day have been used in published trials in children and adolescents for other indications [91], but no dose is proposed here: there are no randomized data in FASD from which a dose could be derived, and stating one would give an operational instruction that the evidence does not support. NAC is discussed as an experimental direction only, to be considered, if at all, in consultation with a specialist.

### 10.12. Fecal Microbiota Transplantation (FMT): Only in Experimental Settings

FMT in adults with alcohol use disorder has shown promising results [31]. In children with FASD, no clinical data are currently available: the intervention is limited to clinical studies only. Position and evidence: This restriction is not a formality, and the reason must be stated explicitly because it is a matter of safety rather than of efficacy. In 2019, the Food and Drug Administration issued a safety communication after two immunocompromised adults developed invasive infection with extended-spectrum beta-lactamase-producing *Escherichia coli* transmitted through investigational FMT; one of them died, and the donor stool had not been screened for these organisms [32]. In 2020, the agency reported six further patients with enteropathogenic or Shiga-toxin-producing *Escherichia coli* infection after FMT, four of whom required hospitalization, together with two additional deaths in patients who had received product from the donor linked to the Shiga-toxin-producing cases [33]. A subsequent alert concerned the possible transmission of mpox. These events occurred in adults under investigational protocols with donor screening in place. No pediatric FASD population has been studied, no donor-screening standard has been validated for this indication, and there is no basis on which a favorable balance of benefit and risk could be assumed in a child. FMT is therefore listed in Table 1 as a tier 4 intervention reserved for clinical trials, and nothing in this review should be read as supporting its use outside that setting.

## 11. Children Below 2 Years of Age: The Early Window (Prenatal and Early Infancy)

Most of the interventions discussed above have RCT validation from 2 years of age upward. For the **first two years of life**, the strategy relies mainly on extrapolation from maternal and general pediatric studies, which, as shown in Section 9.3, is particularly justified in children with FASD because approximately 64% of them belong to the preterm or growth-restricted populations (18.4% preterm and 51.4% small for gestational age, with 5.9% meeting both criteria [62]) in which the interventions have already been validated. A practical map is described:

**Prenatal period.** In any pregnant woman with documented or strongly suspected alcohol consumption during pregnancy: **choline—maternal supplementation of approximately 2 g of choline per day (as choline bitartrate) throughout pregnancy** (level of evidence: RCTs by Jacobson 2018 and Warton 2021) [6,7]; **folates—in accordance with the 2024 position of the Polish Society of Gynecologists and Obstetricians: 400 µg 5-MTHF together with 400 µg folic acid per day in the periconceptional period, started at least 12 weeks before conception, and 800 µg 5-MTHF per day throughout pregnancy and lactation** [10]; **DHA**—at least 200 mg/day in accordance with the EFSA and Polish society consensus; in high-risk situations ≥300 mg/day; **PAE-targeted maternal supplementation—iron, vitamin D, zinc, and B-group vitamins; in a randomized trial in alcohol-exposed pregnancies in Ukraine, multivitamin and mineral supplementation with or without choline was associated with better attentional outcomes in the offspring at preschool age** [11].

**In the breastfeeding woman, the adequate intake of choline is 520 mg/day, derived by adding the roughly 120 mg/day secreted in milk during the first six months of exclusive breastfeeding to the adequate intake for non-lactating women** [92]; **the United States Institute of Medicine sets a corresponding value of 550 mg/day. Infancy up to 6 months: a breastfed child. Breastfeeding is the first pillar of intervention** in a child with PAE for three reasons: 1. breast milk provides HMOs, *Bifidobacterium*, lactoferrin, and SCFAs; 2. breast milk contains choline (phosphatidylcholine and sphingomyelin), DHA, and micronutrients that are deficient in children with FASD; 3. breastfeeding shifts the infant’s microbiome in a direction opposite to PAE-induced dysbiosis.

Supplementation of the breastfeeding mother (continuation of choline, DHA, and vitamins) has a mechanistic basis, but specific RCTs are lacking.

**Infancy up to 6 months: a non-breastfed child.** This is the highest-risk group (including foster-care and adopted children). Strategy: **Infant formula** selected in accordance with the principles described in Section 10.10 (with GOS/FOS or HMOs, adequate DHA + ARA (ratio at least 1:1), and choline); ***L. rhamnosus* GG (ATCC 53103)** at a dose of 5 × 10^9^ CFU/day from the first months of life (strong early-infancy evidence from the Pärtty 2015 study: a 13-year follow-up showed a substantial reduction in the risk of ADHD and ASD) [18]; **Vitamin D** in accordance with Polish recommendations (400–600 IU/day); **in a child who is simultaneously a preterm infant or growth-restricted**, additionally consider enteral choline at 30 mg/kg/day by analogy to the Böckmann protocol [15] (individual decision in a referral center); **monitoring of growth, ferritin, B_12_, and fat-soluble vitamins** every 3 months during the first year.

**Ages 6–24 months: expansion of the diet.** As complementary foods are introduced: natural dietary sources of choline (egg yolk, liver, and salmon: introduced early); natural prebiotics (vegetables, fruits, and whole-grain products where tolerated); continuation of DHA from formula/foods (fatty marine fish 1–2× weekly from 8–9 months); continuation of *L. rhamnosus* GG; **no routine supplementation with sodium butyrate in capsule form** (in the case of gastrointestinal symptoms, referral to a pediatric gastroenterologist).

## 12. A Practical Decision Map: Where to Start in an Individual Child

The following map is presented as a **hypothesis-generating decision framework**, based largely on extrapolation from related pediatric populations and on mechanistic rationale rather than on formally validated clinical guidelines for FASD. Individual steps may be considered by the clinician on a case-by-case basis, following clinical assessment and specialist consultation. Only the T1-level recommendation (RCTs specific to FASD) applies to choline supplementation in the pregnant woman and in the child aged 2–5 years; the remaining steps should be understood as proposals to be validated in prospective clinical studies in the FASD population.

**Step 1.** Baseline assessment (laboratory panel + extended history + perinatal data: gestational age and birth weight).

**Step 2.** Correction of identified deficiencies (iron, vitamin D, zinc, and—where indicated—B_12_).

**Step 3.** Choline: **Pregnant woman with PAE → choline ~2 g/day (bitartrate)**; **child ≥ 2 years** → choline 500–625 mg/day (bitartrate); **infant < 2 years** → optimization through breast milk or a formula containing choline; in preterm/SGA infants, additionally consider 30 mg/kg/day enterally under specialist supervision.

**Step 4.** Omega-3: **Pregnant and breastfeeding woman** → 200–300 mg DHA/day; **infant 0–12 months** → DHA + ARA in formula (ratio ≥ 1:1) or pure microalgal DHA at 100 mg/day, matching the adequate intake of 100 mg DHA/day set for infants and young children up to 24 months [8]; **child ≥ 2 years** → 150–500 mg DHA + EPA/day, DHA:EPA ratio at least 2:1; **child ≥ 6 years with depression** → an EPA ≥ 60% preparation at 250–1000 mg/day.

**Step 5.** In any child with FASD ≥ 2 years with gastrointestinal symptoms: **sodium butyrate** (150–300 mg twice daily in microencapsulated form); in children <2 years: modulation through a formula containing GOS/FOS or HMOs.

**Step 6.** After 4–8 weeks: addition of the probiotic ***L. rhamnosus* GG (ATCC 53103)** (10^9^–10^10^ CFU/day) as a long-term intervention; in infants: from the first months of life at a dose of 5 × 10^9^ CFU.

**Step 7.** Depending on the predominant phenotype: anxiety–depressive symptoms → add a **psychobiotic** (*L. plantarum* PS128 or *L. helveticus* R0052 + *B. longum* R0175) for 8–12 weeks; ADHD-like symptoms → consider a **synbiotic** composed of the *Pediococcus*/*Lactobacillus*/*Leuconostoc* strains with the fibers mentioned in Section 10.8; a severe oxidative picture (e.g., neurological disturbances, a history of seizures) → specialist consultation; no dose of N-acetylcysteine is proposed in this review.

**Step 8.** In the case of predominantly post-dairy abdominal symptoms: targeted diagnostic work-up (lactose breath test, stool lactose, and calprotectin) **before** prescribing lactase; lactase as a symptomatic bridge, not as a replacement for a causal intervention (Section 10.9).

**Step 9.** Monitoring every 3 months: clinical assessment (behavior, sleep, appetite, and gastrointestinal motility); after 6 months, repeat of the laboratory panel; psychometric scales (CBCL, SDQ, and PedsQL) every 6 months.

**Step 10.** Every child with FASD requires parallel care: psychological/psychotherapeutic (FASD-adapted cognitive behavioral therapy); educational (individualized educational program); family (caregiver support and education).

Nutritional and microbiological intervention **does not replace** these elements: it complements them at the biological level.

## 13. A Special Situation: A Child with FASD in Foster Care—Polish Realities

In Polish realities, this is the group with the highest concentration of children with FASD. Practical recommendations for this group are as follows:

1. **FASD diagnostics without waiting for the history**: in any child in foster care with neurodevelopmental difficulties (screening tools: FASDetect, Hoyme 2016 criteria [93]).
2. **Extended nutritional assessment**: ferritin, vitamin D, B_12_, erythrocyte omega-3, weight/length assessed on growth charts, and assessment of motor maturation; particular attention in children with a history of preterm birth or SGA.
3. **Infant formula as the “first line of postnatal intervention”**: with lactose (not to be reduced routinely), GOS/FOS or HMOs, DHA ≥ 20 mg/100 kcal **together with ARA** in an ARA:DHA ratio of at least 1:1, and choline in accordance with current EU recommendations (details of the choice: Section 10.10).
4. ***L. rhamnosus* GG (ATCC 53103) from the first months**: 5 × 10^9^ CFU/day (strongest early-infancy data from the Pärtty 2015 study [18]).
5. **Choline as an expert recommendation in an infant with FASD without access to maternal milk**: as described in Section 10.1.3 (the third-line pathway); an individual decision in a referral center, particularly in children with preterm birth or SGA.
6. **Stability of care as a medical intervention**: educating professionals in the foster-care system that a change of caregiver has a biological dimension (the HPA axis) and not just a psychological one.
7. **Education of foster caregivers**: most foster families lack knowledge about the specifics of FASD and about nutritional interventions; training programs represent an urgent systemic gap.
8. **Cooperation with a clinical dietitian**: in every child with FASD in foster care, regardless of age, because of the high risk of malnutrition and specific micronutrient deficiencies.

### Implementation Challenges of the Protocol in the Polish Foster-Care System

The above recommendations require consideration of real regulatory and organizational barriers.

**Regulatory categorization of preparations.** The individual components of the protocol belong to different legal categories in Poland and the EU, which determines the access and financing pathway. GOS/FOS prebiotics and human milk oligosaccharides (HMOs) are permitted as components of infant formulas under EU Regulation 2016/127 [14]: their availability is widespread. DHA and ARA are likewise permitted as formula components and are available as dietary supplements. Bovine lactoferrin at 100 mg/day was studied in very-low-birth-weight neonates, in whom it reduced late-onset sepsis; the endpoint was infectious rather than neurodevelopmental, and the product was supplied by the manufacturer [17]. The current ESPGHAN position lists lactoferrin among supplements for which there is insufficient evidence to support routine use in preterm infants [13], and this proposal does not override that assessment. Bovine lactoferrin is available in Poland as a component of selected FSMPs for preterm infants and as a dietary supplement; the FSMP category enables reimbursement by the National Health Fund (NFZ, *Narodowy Fundusz Zdrowia*) upon documented medical indication. Phosphatidylcholine and choline in physiological forms are present in standard infant formulas, whereas dedicated pediatric preparations (alpha-GPC and egg-PC) have limited availability. Alpha-glycerophosphocholine is registered in Poland mainly as a dietary supplement for adults; **the absence of preparations dedicated to infants** represents a real regulatory barrier. *Lactobacillus rhamnosus* GG is available as a dietary supplement and as a component of some formulas.

**Financing pathways.** In the Polish system, there are three main pathways: (i) NFZ reimbursement is available for preparations in the FSMP category with a documented medical indication (preterm birth, intrauterine growth restriction, and feeding intolerance); this requires a physician’s prescription. A recommendation is active referral of children with FASD to a pediatric gastroenterologist or neonatologist to establish an FSMP indication. (ii) The care and educational institution’s budget covers standard feeding; additional preparations require separate justification and may exceed the budgetary standard. (iii) The foster family’s own funds can be a real burden, particularly problematic in kinship foster families and with lower benefits.

**Procurement procedures.** Care and educational institutions typically operate on the basis of pre-negotiated contracts with a food supplier or pharmacy. Introducing a new preparation requires a clinical justification accepted by the referring physician, administrative agreement with the governing body (county self-government), and a procurement procedure, for larger institutions often via public tender, which prolongs the time to intervention.

**Staff competencies.** Standard training of nursing staff in Polish care institutions does not include detailed knowledge of nutrition of infants with FASD or of specialist preparations. Introduction of the protocol requires a parallel educational program for staff (dosing, monitoring, and recognition of adverse effects).

**Policy and systemic recommendation.** The above barriers are structural and cannot be resolved by individual clinician efforts alone. Full implementation of the proposed protocol requires (i) the establishment of **national guidelines** for the feeding of infants with suspected FASD in foster care, developed by the Polish Pediatric Society (PTP, *Polskie Towarzystwo Pediatryczne*) in cooperation with the Polish Neonatal Society and the National Consultant; (ii) extension of FSMP reimbursement to the indication “suspected FASD with unavailable natural feeding”; (iii) **educational programs** for staff of care and educational institutions; and (iv) establishment of a nutritional standard in force in Polish care institutions that takes into account the high prevalence of FASD.

The individual intervention of a pediatric clinician can, in turn, include (i) routine consideration of FASD in the differential diagnosis of children admitted to foster care; (ii) referral to a pediatric gastroenterologist to establish an FSMP indication; and (iii) recommendation of an infant formula with optimal supplementation with GOS/FOS prebiotics, DHA + ARA, and lactoferrin: this is an intervention that is practically feasible in every Polish institution already today.

## 14. Limitations, Future Directions, and Conclusions

### 14.1. Limitations

The present article is a narrative, not a systematic, review. Most of the proposed interventions have no RCTs specific to FASD: we rely on extrapolation from populations of healthy infants, preterm infants, growth-restricted infants, children with IBS/IBD, and children with ADHD/ASD and on a strong mechanistic rationale. The doses and duration of interventions require validation in prospective clinical trials. **None of the proposed interventions is registered in Poland or the European Union as a treatment for FASD**; they are used as dietary supplements, foods for special medical purposes, or components of infant foods. All recommendations in this work are of the nature of an expert proposal and require individualization in consultation with a specialist. The work does not refer to trade names of specific preparations.

**A note on the evidence tiers used in** Table 1**.** In this review, we explicitly distinguish three levels of evidence for each intervention: **Tier 1**—evidence from RCTs specific to FASD (currently only choline supplementation: Wozniak et al. 2015, 2020 [20,22]; Jacobson et al. 2018 [6]; Warton et al. 2021 [7]); **Tier 2—evidence from RCTs extrapolated from related pediatric populations (probiotics per Pärtty et al.** [18]**; prebiotics per Moro et al.** [27]**; DHA and ARA per the European Academy of Paediatrics and Child Health Foundation position** [9]**);** and **Tier 3**—mechanistic rationale and expert opinion without RCT validation (bovine colostrum, sodium butyrate >24 months, and iron and B_12_ at confirmed deficiency). This distinction is reflected in the “Strength of evidence” column of Table 1 and is signposted in the discussion text for each therapeutic recommendation.

**Methodological limitations of the review.** Restricting inclusion to publications in English and Polish may omit important data in other languages; we have attempted to minimize this risk by relying on meta-analyses and systematic reviews with a broad linguistic scope. Extending the search strategy to include Embase and Web of Science during the review process did not reveal publications that would change the fundamental conclusions but did provide several supplementary items. CINAHL (nursing) and PEDro (physiotherapy) were not included, as these are outside the thematic scope of the work.

### 14.2. Future Directions

- **RCTs of individual interventions in children with FASD**: sodium butyrate, *L. rhamnosus* GG, omega-3, the choline + omega-3 combination, and the psychobiotic *L. plantarum* PS128;
- **RCTs of interventions in infants with FASD**: the least studied window and the greatest potential benefit, particularly in the subgroup of preterm and growth-restricted infants;
- **A Polish multicenter study** extending the Okulicz-Kozaryn protocol to the whole population, with metagenomic analysis of the microbiota in mother–child pairs;
- **Metabolomic profiling** (kynurenine/tryptophan and SCFAs) as a biomarker distinguishing FASD from ADHD;
- **Studies of integrated protocols**: nutrition + psychotherapy + education as a package;
- **Studies of the effects of co-exposure**—alcohol + tobacco, alcohol + cannabis, alcohol + opioids—on the microbiota and behavioral phenotype;
- **Optimization of interventions in children in foster care** as the population of greatest need.

### 14.3. Conclusions

Fetal alcohol spectrum disorder is not a disorder confined solely to the brain: a growing body of evidence suggests that an important role in its pathophysiology is also played by the **dialogue between the gut and the brain**, established in fetal life and sustained through a cycle of dysbiosis → LPS → neuroinflammation → serotonin deficit → quinolinic acid → HPA-axis dysregulation. This peripheral component may substantially contribute to the high rates of depression, self-injurious behavior, and suicide attempts observed in children with FASD, as a complementary mechanism, not as a replacement for the classical neurotoxicity of ethanol.

In the Polish context, the problem is amplified by the systematic underestimation of prenatal exposure (the IMiDz study: 50% of women drink during pregnancy; a sevenfold gap between self-reporting and EtG), by the overlap between the FASD and ADHD phenotypes, and by additional clinical burdens: prenatal co-exposure to nicotine and other substances, loss of vertical microbiota transmission in children left in foster care, and the over-representation of preterm and growth-restricted infants (51.4% SGA, 18.4% preterm among children with FASD).

The structured four-stage protocol we propose—assessment → correction of deficits → microbiota modulation → targeted neuroprotective interventions—is offered as a **hypothesis-generating clinical framework** that a clinician may consider on the basis of the child’s age and risk profile. **In a pregnant woman with PAE, one may consider starting with maternal choline ~2 g/day and DHA ≥ 300 mg/day (level of evidence: RCT specific to FASD—T1). In an infant with FASD who is not breastfed, one may consider a formula with lactose, GOS/FOS or HMOs, DHA and ARA at an ARA:DHA ratio between 0.5 and 2** [13]**, and choline, together with *L. rhamnosus* GG from the first months (level of evidence: extrapolation from RCTs in preterm infants and in healthy infants—T2); in preterm and growth-restricted infants, one may additionally consider enteral choline at 30 mg/kg/day by analogy to the randomized neonatal data (T3, and expressly contrary to the current ESPGHAN position, which does not recommend routine choline supplementation in preterm infants** [13]**). In a child ≥2 years, one may consider choline 500–625 mg/day (T1), sodium butyrate 150–300 mg twice daily (T2, extrapolated from pediatric IBS), omega-3 with DHA dominance (T2), *L. rhamnosus* GG (T2), and—depending on the phenotype (depression vs. ADHD)—the appropriate psychobiotic or synbiotic (T3, mechanistic).** All these interventions have a mechanistic rationale and—for most—RCTs in related pediatric populations; however, **none of the proposals other than choline supplementation in the pregnant woman and in the child aged 2–5 years has formal validation in RCTs in the FASD population**. Obtaining such RCTs is the most important research priority for the coming decade. Until then, each therapeutic decision requires individualized assessment, specialist consultation, and consideration of the available clinical alternatives.

Primary prevention—total abstinence during pregnancy—remains the overriding priority. The Polish IMiDz data show, however, that the current model of educating pregnant women **fails systemically**, and children who have already been born with FASD deserve care that reflects the current body of knowledge on the gut–brain axis.

## Figures and Tables

**Table 1:** **Proposed staged nutritional and microbiological supplementation in children with FASD—hierarchy of evidence by tiers.** CLINICAL NOTE. None of the interventions listed in this table is registered in Poland or in the European Union as a treatment for FASD. The table presents a protocol proposal grounded in a hierarchy of scientific evidence. Each intervention requires individualized assessment and a decision by a specialist (pediatrician, neonatologist, pediatric gastroenterologist, or clinical dietitian). The protocol is hypothesis-generating and is not a clinically validated guideline.

Substance/Strain	Age Window	Proposed Dose	Evidence Tier	Key Publication
**PRENATAL PERIOD—intervention in the pregnant woman with PAE**				
Choline (bitartrate)	Entire pregnancy	~2 g/day orally	T1—RCT in FASD	Jacobson 2018 [6]; Warton 2021 [7]
DHA (docosahexaenoic acid)	Entire pregnancy + lactation	200–300 mg/day; in PAE preferably 300 mg	T2—EFSA consensus	EFSA 2013 [8]; Koletzko 2020 [9]
Folic acid	≥3 months before pregnancy + first trimester	400 µg/day preconception; 800 µg/day in first trimester	T2—standard of care	PTGiP recommendations [10]
Multimicronutrient (Fe, vitamin D, zinc, B-group)	Entire pregnancy	Individualized to documented deficits	T3—expert opinion + mechanism	Kable 2022 [11]; Helfrich 2018 [12]
**AGE 0–12 MONTHS—priority: breastfeeding; formula as second-line**				
Breastfeeding (with maternal supplementation)	0–6 months (exclusive), up to 24 months (complementary)	First-line; continuation of maternal choline 520 mg/day (EFSA adequate intake in lactation) + DHA 300 mg/day	T1—gold standard	WHO; ESPGHAN 2022 [13]
Fortified formula (choline + DHA + ARA + prebiotics)	0–12 months (when breastfeeding is unavailable)	Formula meeting EU 2016/127 [14]: choline 25–50 mg/100 kcal, DHA ≥ 20 mg/100 kcal, ARA:DHA ratio 0.5–2 per ESPGHAN 2022 [13]	T2—extrapolation from ESPGHAN 2022 [13]	EU Regulation 2016/127 [14]; ESPGHAN 2022 [13]
Choline—additional supplementation in preterm/SGA infants	Preterm and SGA infants with FASD	30 mg/kg/day enterally for 10 days (Böckmann protocol); all four choline preparations tested were TMAO-neutral	T3—contrary to current ESPGHAN position	Böckmann 2026 [15]; Böckmann 2023 [16]; ESPGHAN 2022 [13] does NOT recommend routine choline supplementation in preterm infants
Bovine lactoferrin	Preterm and growth-restricted infants (0–6 months)	100 mg/day orally (in formula or as FSMP)	T3—contrary to current ESPGHAN position	Manzoni 2009 [17]; ESPGHAN 2022 [13] finds insufficient evidence for routine use
Lactobacillus rhamnosus GG (ATCC 53103)	From 1 month of age	1 × 10^9^–1 × 10^10^ CFU/day orally	T2—RCT in the infant population	Pärtty 2015 [18]; van den Akker 2020 [19]
**AGE 1–2 YEARS—child with FASD**				
Choline	1–2 years	Natural sources (egg yolk, salmon, liver); no direct RCT data	T3—evidence gap	Wozniak 2015 [20] from 2.5 years; Wozniak 2013 [21]
DHA + ARA (continuation)	12–24 months	DHA 100 mg/day (EFSA adequate intake to 24 months); ARA in an equivalent amount	T2—ESPGHAN 2022 [13]	ESPGHAN 2022 [13]; Koletzko 2020 [9]
*L. rhamnosus* GG	Continuation	1 × 10^9^–1 × 10^10^ CFU/day	T2—RCT in the infant population	Pärtty 2015 [18]
**AGE 2–5 YEARS—preschool child with FASD (RCT window)**				
Choline (bitartrate)	2.5–5 years	500–625 mg/day for ≥ 9 months	T1—RCT in FASD	Wozniak 2015, 2020 [20,22]
Sodium butyrate (microencapsulated)	≥ 2 years	150–300 mg × 2/day with meals	T2—pediatric IBS RCT	Cristofori 2025 [23]; Bakshi & Mishra 2025 [24]
Omega-3 (DHA-dominant)	≥ 2 years	150–500 mg/day DHA + EPA (DHA:EPA ratio ≥ 2:1)	T2—extrapolation from ADHD	Lien 2018 [25]
*L. rhamnosus* GG	Continuation	1 × 10^9^–1 × 10^10^ CFU/day	T2—extrapolated RCT	Pärtty 2015 [18]; Skott 2020 [26]
GOS/FOS prebiotics (galacto-oligosaccharides/fructo-oligosaccharides)	Entire pediatric population	In fortified formulas (infancy); in older children 1–2 g/day—expert opinion (no trial extends beyond infancy)	T2—RCT in the infant population	Moro 2002 [27]; Arslanoglu 2008 [28]
**AGE 5–18 YEARS—school-age child/adolescent with FASD**				
Choline	5–18 years	500–1000 mg/day (expert suggestion; narrower therapeutic window)	T2—RCT with limited effect	Wozniak 2015 [20]
Sodium butyrate	Selected phenotypes	300 mg × 2/day	T3—mechanistic	Cristofori 2025 [23]; Recharla 2023 [29]
Psychobiotic (L. plantarum PS128)	Depressive/anxiety symptoms	3 × 10^10^ CFU twice daily (6 × 10^10^ CFU/day) for 4 weeks (trial regimen)	T3—RCT extrapolated from ASD	Liu 2019 [30] (ASD)
Synbiotic (probiotic + prebiotic)	ADHD-like symptoms	Per formulation	T3—RCT ADHD extrapolation	Skott 2020 [26]
**TARGETED INTERVENTIONS—specific phenotypes**				
N-acetylcysteine (NAC)	≥ 3 years (experimental direction)	No dose proposed—no FASD-specific data; pediatric doses in other indications are reported in the main text (Section 10.11)	T3—limited RCT data	Literature in main text
FMT (fecal microbiota transplantation)	Experimental settings only	Not for routine use	T4—experimental	Wolstenholme 2024 [31]; FDA safety communications [32,33]

LEGEND—EVIDENCE TIERS. T1 (green)—Evidence-based for FASD: evidence from RCTs in the FASD population, or a gold standard (e.g., breastfeeding). T2 (yellow)—Extrapolation from related pediatric populations: RCTs in preterm infants, growth-restricted infants, children with IBS/IBD, or ADHD/ASD. T3 (orange)—Mechanistic rationale + expert opinion: strong pathophysiological basis, limited FASD-specific RCT evidence; rows marked “contrary to current ESPGHAN position” are declared extrapolations that depart from the cited society position, addressing documented or strongly suspected PAE rather than routine supplementation of unselected infants. T4 (red)—Experimental only: reserved for clinical trials. Abbreviations: PAE—prenatal alcohol exposure; SGA—small-for-gestational-age; FSMP—food for special medical purposes; CFU—colony-forming units; ATCC—American Type Culture Collection; DHA—docosahexaenoic acid; ARA—arachidonic acid; EPA—eicosapentaenoic acid; GOS/FOS—galacto-oligosaccharides/fructo-oligosaccharides; FMT—fecal microbiota transplantation; NAC—N-acetylcysteine; RCT—randomized controlled trial; PTGiP—Polish Society of Gynecologists and Obstetricians; ESPGHAN—European Society for Paediatric Gastroenterology, Hepatology and Nutrition; EFSA—European Food Safety Authority; WHO—World Health Organization.

## Data Availability

No new data were created or analyzed in this study. Data sharing is not applicable to this article.
